# Molecular landscape of prostate cancers with clival metastases

**DOI:** 10.1093/oncolo/oyag074

**Published:** 2026-03-04

**Authors:** Pornlada Likasitwatanakul, Steven M Blinka, Jabra G Zarka, Georges Gebrael, Emily Weg, Ossian Longoria, Joseph A Moore, Adam Sharp, Johann de Bono, Cora N Sternberg, Neeraj Agarwal, Umang Swami, Jacob J Orme, Michael T Schweizer, Lindsey Sloan, Justin H Hwang, Emmanuel S Antonarakis

**Affiliations:** Department of Medicine, University of Minnesota-Twin Cities, Minneapolis, MN 55455, United States; Department of Medicine, Siriraj Hospital, Mahidol University, Bangkok, 10700, Thailand; Clinical Research Division, Fred Hutchinson Cancer Center, Seattle, WA 98109, United States; Division of Hematology and Oncology, University of Washington School of Medicine, Seattle, WA 98195, United States; Department of Oncology, Mayo Clinic, Rochester, MN 55905, United States; Division of Oncology, University of Utah Huntsman Cancer Institute, Salt Lake City, UH 84112, United States; Department of Medical Oncology, Weill Cornell Medicine and the Englander Institute for Precision Medicine, New York, NY 10021, United States; Division of Clinical Studies, The Institute of Cancer Research, London, SM2 5GP, United Kingdom; Drug Development Unit and Prostate Cancer Targeted Therapy Group, The Royal Marsden NHS Foundation Trust, London, SM2 5PT, United Kingdom; Department of Radiation Oncology, University of Minnesota-Twin Cities, Minneapolis, MN 55455, United States; Division of Clinical Studies, The Institute of Cancer Research, London, SM2 5GP, United Kingdom; Drug Development Unit and Prostate Cancer Targeted Therapy Group, The Royal Marsden NHS Foundation Trust, London, SM2 5PT, United Kingdom; Division of Clinical Studies, The Institute of Cancer Research, London, SM2 5GP, United Kingdom; Drug Development Unit and Prostate Cancer Targeted Therapy Group, The Royal Marsden NHS Foundation Trust, London, SM2 5PT, United Kingdom; Department of Medical Oncology, Weill Cornell Medicine and the Englander Institute for Precision Medicine, New York, NY 10021, United States; Division of Oncology, University of Utah Huntsman Cancer Institute, Salt Lake City, UH 84112, United States; Division of Oncology, University of Utah Huntsman Cancer Institute, Salt Lake City, UH 84112, United States; Department of Oncology, Mayo Clinic, Rochester, MN 55905, United States; Clinical Research Division, Fred Hutchinson Cancer Center, Seattle, WA 98109, United States; Division of Hematology and Oncology, University of Washington School of Medicine, Seattle, WA 98195, United States; Department of Radiation Oncology, University of Minnesota-Twin Cities, Minneapolis, MN 55455, United States; Masonic Cancer Center, University of Minnesota-Twin Cities, Minneapolis, MN 55455, United States; Department of Medicine, University of Minnesota-Twin Cities, Minneapolis, MN 55455, United States; Masonic Cancer Center, University of Minnesota-Twin Cities, Minneapolis, MN 55455, United States; Department of Medicine, University of Minnesota-Twin Cities, Minneapolis, MN 55455, United States; Masonic Cancer Center, University of Minnesota-Twin Cities, Minneapolis, MN 55455, United States

**Keywords:** prostate cancer, clivus bone, clival metastasis, next-generation sequencing

## Abstract

**Background:**

Clival metastases are a rare and clinically aggressive manifestation of advanced prostate cancer, associated with cranial nerve palsy and poor survival. The molecular features of prostate cancers giving rise to clivus metastases remain unknown.

**Patients and methods:**

We performed a multi-center retrospective study across six institutions, identifying prostate cancer patients with radiographically confirmed clival metastases and available next-generation sequencing (NGS) data. Baseline characteristics and clinical outcomes were collected. Genomic alterations from tissue- and/or blood-based assays were aggregated at the patient level and compared with a publicly available metastatic castration-resistant prostate cancer (mCRPC) cohort (SU2C/PCF).

**Results:**

Fifty-nine patients with clival metastases contributed 87 molecular assays. More than half of patients had Gleason grade group 5 cancer and presented with *de novo* metastatic (M1) disease. The median interval from initial prostate cancer diagnosis to clival metastasis was 71.4 months (95% CI, 42.0-101.7), while median overall survival following clival involvement was only 15.3 months (95% CI, 6.9-22.8). Compared with the SU2C/PCF mCRPC cohort, clival metastases showed significant enrichment of *BRAF* and *CHEK2* alterations as well as homologous recombination repair (HRR) with relative depletion of AR-related, PI3K pathway, and G2–M pathway alterations.

**Conclusion:**

Prostate cancers giving rise to clival metastases exhibit a distinct molecular profile enriched for DNA damage–repair and RAF kinase alterations, suggesting unique metastatic biology and potential therapeutic vulnerabilities.

Implications for Practice Clival metastases are a rare but serious complication of advanced prostate cancer because of their location near critical brainstem and cranial nerve structures and their historically poor prognosis. This study shows that prostate cancers spreading to the clivus often carry specific genetic changes, particularly defects in DNA repair pathways and the RAF kinase pathway. These findings suggest that clival metastases may respond differently to treatment than other metastatic sites and may be vulnerable to targeted therapies such as PARP inhibitors or MAPK pathway-directed agents. Recognizing this unique molecular profile may help clinicians consider precision-based treatment strategies to improve care for this patient population.

## Introduction

Prostate cancer is the most common noncutaneous malignancy in men globally.^1^^,^^2^ Although prostate cancer is often indolent, with an excellent 5-year overall survival (OS) of 98%, outcomes decline sharply once metastatic disease develops: patients with metastatic PC have a 5-year OS of only 38%.^3^^,^^4^ The bone is the most frequent site of metastasis, occurring in up to 80% of patients with stage IV disease,^5^ and a higher osseous metastatic burden correlates with worse survival.^6^ Symptoms of bone metastasis may include pain, fracture, hypercalcemia, and spinal cord and nerve compression, which not only affects quality of life but also contributes to morbidity and mortality.

Prostate cancer bone metastases typically involve the pelvis, spine, and appendicular skeleton, with calvarial spread being rarer. The clivus, a bone in the central part of the skull base, is an uncommon metastatic site, and clivus tumors represent less than 1% of all cranial metastases.^7^^,^^8^ Critical structures in or near the clivus include the brainstem, cranial nerves (CN) V and VI, basilar artery, and internal carotid arteries. CN VI palsy was observed in approximately half of patients with clival metastasis.^7^^,^^9^ Additional presentations include other CN palsy, headaches, facial pain or numbness, photophobia, or vertigo and sometimes may be asymptomatic.^7^^,^^9^^,^^10^ Despite timely management with radiation or surgery, prognosis historically remains poor.^7^^,^^10^

As of 2025, only 67 cases of clival metastases from solid tumors have been reported in the world literature, with prostate cancer representing the most common primary tumor giving rise to clivus involvement (34%, 23/67).^7^^,^^10^ The median survival of patients with clival metastases receiving either radiation or surgery ranged from 6 to 15 months.^7^^,^^9^^,^^10^ In the prior literature, many of these patients were treated with forms of radiation therapy, including gamma-knife radiosurgery, external beam radiotherapy, or whole brain radiation, and a minority underwent surgical resection.^7^^,^^10^ Notably, none of the existing reports have described the genomic features of cancers metastasizing to the clivus bone.

Given the rarity and high mortality of clival metastases, molecular characterization may offer new insights into their biology. Here, we present the largest multi-center molecular dataset to date of patients with prostate cancer and clival metastases.

## Patients and methods

### Study design and patient selection

This was a multi-center, retrospective cohort study conducted across six institutions, including the University of Minnesota, the Mayo Clinic, Fred Hutchinson Cancer Center, the University of Utah, New York-Presbyterian/Weill Cornell Medical Center, and the Institute of Cancer Research/Royal Marsden NHS Foundation Trust in London. Electronic medical records were reviewed to identify patients with prostate cancer who developed radiographic evidence of clival metastases between January 2015 and November 2025. This retrospective study was performed under Institutional Review Board (IRB) approval at each participating site.

Patients were included if they met the following criteria: (1) histologically confirmed prostate cancer; (2) radiographically confirmed metastases to the clivus bone by CT scan, PET scan, or bone scan; and (3) availability of multi-gene next-generation sequencing (NGS) results from either tumor tissue or blood-based assays.

### Data collection

Clinical variables were extracted from electronic health records and included: demographics (age, race, ethnicity), tumor pathology and Gleason grades, clinical presentation at the time of clival metastasis, pathological or clinical staging at initial diagnosis, prostate-specific antigen (PSA) values at diagnosis and at time of clivus involvement, radiologic imaging findings, and systemic and local therapies received (androgen deprivation therapy, chemotherapy, radiation, surgery, or targeted therapy). The AJCC Cancer Staging 8th Edition was used to define tumor stages at initial diagnosis.

### Molecular profiling

NGS profiling was performed as part of routine clinical care (or for research) across participating institutions using validated clinical-grade (or research-grade) multi-panel or whole-exome genomic tests. Tissue-based sequencing assays performed in the study included FoundationOne CDx (*n* = 16), Tempus xT (*n* = 12), Caris Life Science (*n* = 7), and internal institutional assays (FHCC, *n* = 15; ICR, *n* = 4). Blood-based ctDNA sequencing assays performed in the study included Guardian 360 (*n* = 22), Foundation Liquid CDx (*n* = 7), Caris Assure (*n* = 3), and Tempus xF (*n* = 1). Molecular results were extracted from clinical reports. The following genomic alterations were included in the analysis when classified as pathogenic or likely pathogenic by the reporting laboratory: missense mutations, in-frame mutations, truncating mutations (frameshift and nonsense mutations), structural variants (gene fusions and rearrangements), splice-site variants, copy number alterations (amplifications or deletions), and tumor mutational burden (TMB, mut/Mb). Additional molecular data include microsatellite instability (MSI) status and DNA mismatch repair (MMR) status. Alteration frequencies in our clivus metastasis cohort were compared with those of the StandUp2Cancer/PCF Dream Team (SU2C) metastatic castration-resistant prostate cancer (mCRPC)^11^ cohort and those of the Stopsack et al. metastatic hormone-sensitive prostate cancer (mHSPC)^12^ obtained from the cBioPortal platform. Only driver alterations were included in genomic analyses.

### Statistical analysis

Descriptive statistics were used to summarize clinical characteristics and genomic alterations. Continuous variables were reported as medians and ranges (minimum and maximum), and categorical variables were expressed as counts and percentages. Hazard ratios (HR) were calculated using the Cox proportional hazards model, and *P*-values were calculated using the log-rank test. Molecular data were visualized with an Oncoprint plot generated with the *ComplexHeatmap* package in R.^13^ Fisher’s exact test and Chi-square test were used to compare categorical variables across groups, with statistical significance defined as a two-sided α = 0.05.

## Result

### Clinical characteristics

We assembled the largest cohort of clival metastases reported to date through a retrospective review of prostate cancer cases across six institutions, identifying 59 patients and 87 NGS-sequenced samples (some patients provided more than one tumor DNA sample). Among all patients, 86% (*n* = 51) were Caucasian, 5% (*n* = 3) were African American, 2% (*n* = 1) were Asian, and 7% (*n* = 4) had unknown race. Patient demographics are summarized in Table 1. The median age at prostate cancer diagnosis was 63 years, and the median age at clival metastasis diagnosis was 70 years.

**Table 1.** Demographic data of patients with clival metastasis.

	Median (range)	
**Age**		
** Age at diagnosis (years)**	63.1 (43.0, 85.4)	
** Age at clival metastasis (years)**	69.8 (52.9, 88.6)	
**Baseline PSA**		
** PSA at diagnosis (ng/mL)**	83.8 (2.6, 5375)	
** PSA at clival metastasis (ng/mL)**	80.6 (0, 3157)	
**Initial staging**	***N* = 59**	**%**
** Stage I**	0	0.0
** Stage II**	6	10.2
** Stage III**	12	20.3
** Stage IV-N1**	4	6.8
** Stage IV-M1**	37	62.7
**Tissue types used for NGS**		
** mHSPC**	19	32.2
** mCRPC**	40	67.8
**Metastasis sites at the time of clivus met**		
** Bone**	57	96.6
** Lymph nodes**	27	45.8
** Liver**	8	13.6
** Brain parenchyma**	5	8.5
** Lung**	5	8.5
** Bladder**	3	5.1
**Pathology (from primary tumor)**		
** Adenocarcinoma**	57	96.6
** Neuroendocrine**	2	3.4
**Gleason grade**		
** Grade group 1 (3 + 3)**	0	0.0
** Grade group 2 (3 + 4)**	2	3.4
** Grade group 3 (4 + 3)**	12	20.3
** Grade group 4 (4 + 4)**	0	0.0
** Grade group 5 (9 − 10)**	33	55.9
** Not determined**	12	20.3
**Symptoms at clival metastasis**		
** Symptomatic (cranial nerve palsies)**	16	27.1
** Asymptomatic**	43	72.9
**Clival-targeted treatment**		
** Radiation**	20	33.9
** Surgery**	0	0.0
** Systemic treatment alone**	39	66.1

Abbreviations: mCRPC, metastatic castration-resistant prostate cancer; mHSPC, metastatic hormone-sensitive prostate cancer; NGS, next-generation sequencing; PSA, prostate-specific antigen.

Nearly 70% of patients with clival metastases initially presented with *de novo* stage IV disease (M1: 62.7%; N1: 6.8%), including 9 patients with clival metastasis at diagnosis. An additional 20% presented with stage III disease and 10% with stage II disease; no patients were diagnosed at stage I. The majority of the patients were mCRPC (*n* = 40, 68%); the rest were metastatic hormone-sensitive prostate cancer (mHSPC) (*n* = 19, 32%). Pathology was predominantly prostate adenocarcinoma (96.6%), with 55.9% harboring Gleason grade group 5 disease.

Initial treatment for mHSPC included androgen deprivation therapy (91.5%), abiraterone (32.2%), docetaxel (28.8%), enzalutamide (13.6%), and other agents. Subsequent lines of therapy included docetaxel (*n* = 25), enzalutamide (*n* = 18), abiraterone (*n* = 15), Lutetium Lu-177 PSMA-617 (*n* = 13), cabazitaxel (*n* = 13), carboplatin (*n* = 10), investigational agents (*n* = 9), sipuleucel-T (*n* = 4), radium-223 (*n* = 4), and others. Overall, this cohort reflects patients with high-grade mCRPC who had undergone multiple lines of systemic therapy before the development of clival metastases, although 15% presented with clivus metastases.

### Clinical outcomes

**Figure 1.**
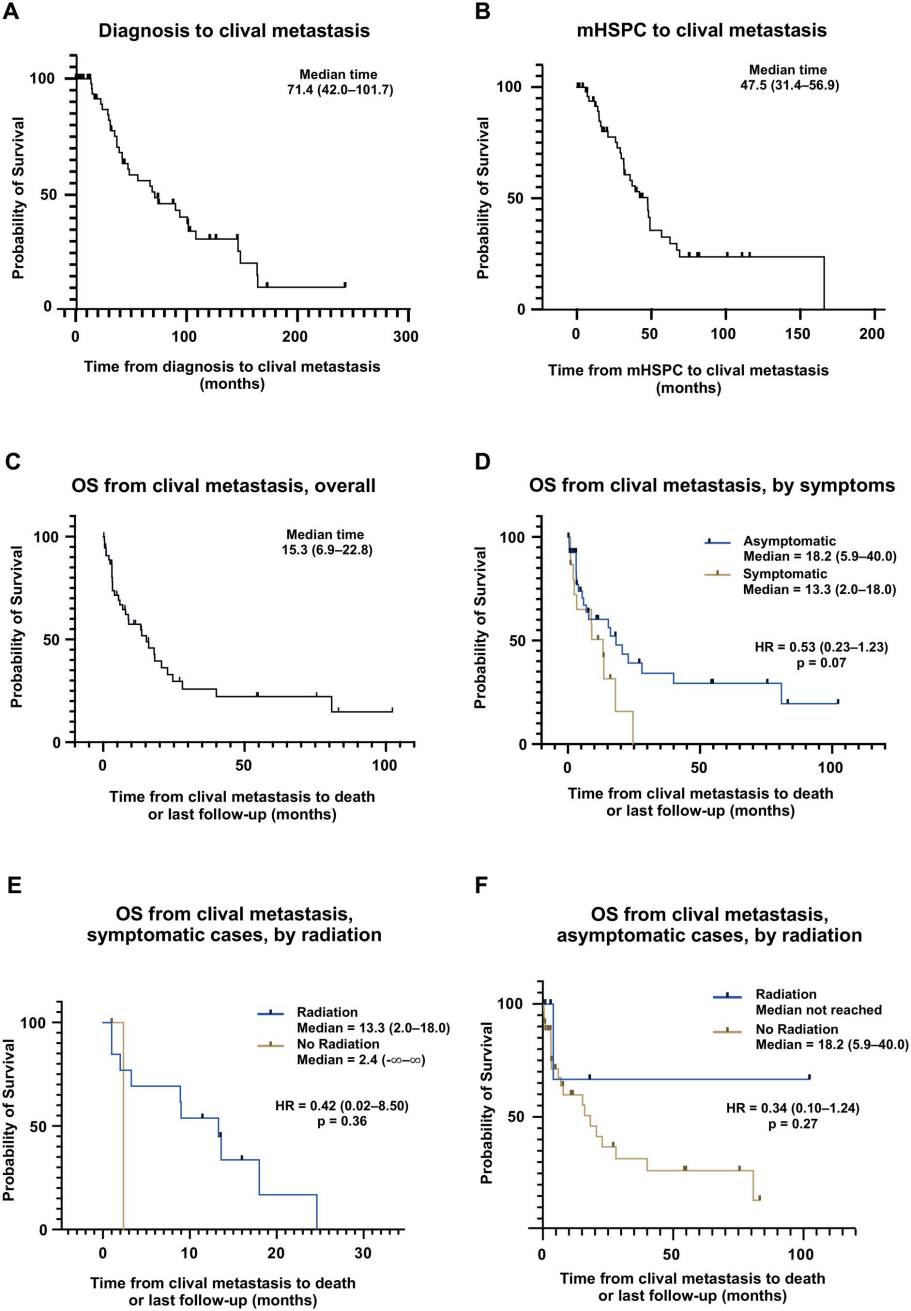
Kaplan–Meier curves depicting: Time from initial diagnosis to development of clival metastases (A); Time from metastatic hormone-sensitive prostate cancer (mHSPC) to clival metastases (B); Overall survival (OS) from diagnosis of clival metastases for all patients (C); OS stratified by the presence or absence of clival-related symptoms (D), OS in symptomatic cases (E) and asymptomatic cases (F) stratified by receipt of radiation therapy. Dashes indicate censored patients alive at the last follow-up.

Clival metastasis portends a poor prognosis.^7^^,^^9^^,^^10^ The median time from prostate cancer diagnosis to the development of clival metastases was 71.4 months (Figure 1A; 95% CI, 42.0-101.7), while the median time from mHSPC to clival metastases was 47.5 months (Figure 1B; 95% CI, 31.4-56.9). At the time of clival involvement, nearly all patients (96.6%) had additional bone metastases, while 13.6% had liver metastases, 8.5% had parenchymal brain metastases, and 8.5% had lung metastases (Table 1).

A total of 27.1% of patients (*n* = 16) presented with clival-related symptoms, whereas 72.9% (*n* = 43) remained asymptomatic. Reported symptoms included diplopia (*n* = 10), facial numbness (*n* = 5), headache (*n* = 4), facial weakness (*n* = 2), hearing impairment (*n* = 2), and dysphonia (*n* = 1). All symptomatic patients except two received radiotherapy; four asymptomatic patients also underwent clivus-directed radiation treatment. No patients underwent surgical intervention for clival metastases.

The median overall survival (OS) following diagnosis of clival metastases was 15.3 months overall (Figure 1C; 95% CI, 6.9-22.8). When stratified by symptom status, symptomatic patients demonstrated worse OS compared with asymptomatic patients (HR, 0.53; 95% CI, 0.23-1.23), although the difference did not reach statistical significance (*P* = .07) (Figure 1D). Notably, all patients with symptomatic clival metastases were dead by 24 months. Patients who received radiation therapy tended to have longer OS in both symptomatic cases (Figure 1E, HR = 0.42; 95% CI, 0.02-8.50) and asymptomatic cases (Figure 1F, HR = 0.34; 95% CI, 0.10-1.24), although this did not reach statistical significance. Overall, these findings highlight that clival metastases typically arise late in the disease course and are associated with limited survival, especially in patients with symptoms related to clivus involvement. Radiation was associated with extended OS regardless of patient symptoms, a finding that was corroborated here.

### Molecular landscape

**Figure 2.**
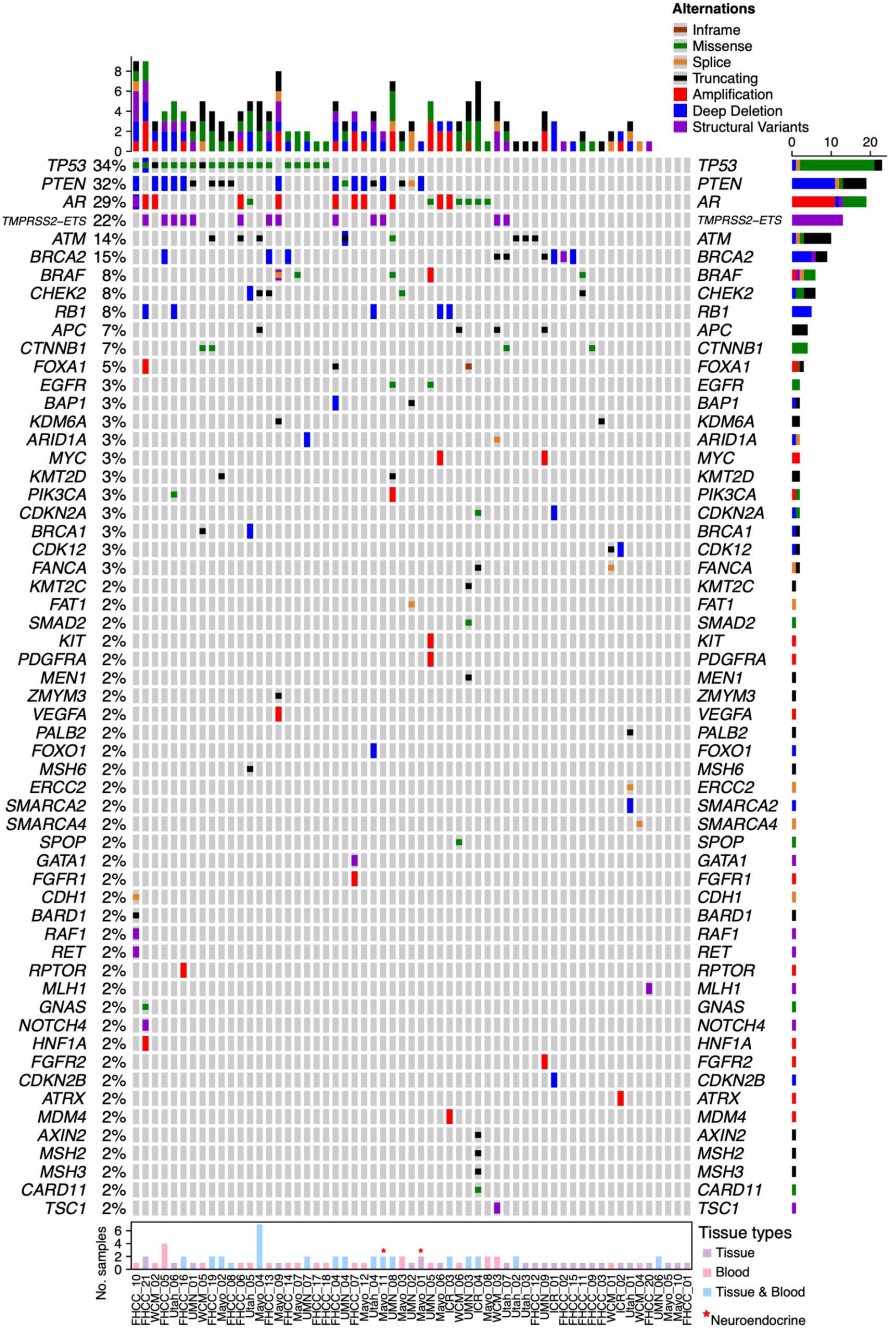
OncoPrint Figure summarizing genomic alterations among all patients with clival metastases (*n* = 59). The top and right panels display bar plots indicating the number and types of alterations per patient and per gene, respectively. The left panel shows the percentage of samples harboring alterations in each gene. The bottom panel depicts the number of samples contributed by each patient. Tissue types are color-coded: tissues (purple), blood (pink) and both (blue). * Denotes neuroendocrine carcinoma; the rest were adenocarcinoma.

Our study is the first to comprehensively characterize the molecular features of prostate cancers giving rise to clival metastases across cancers. A total of 87 molecular assays were analyzed, including 42 tissue specimens and 45 blood-based ctDNA assays from 59 prostate cancer patients. Thirty-one patients (52.5%) had samples collected at diagnosis, and 33 (55.9%) had samples collected at or later than the time of clival metastases (Table S1). Among tissue samples, 19 originated from prostate lesions, 10 from bone metastases, eight from lymph nodes, three from liver, and two from other metastatic sites (Table S2). All molecular results from each patient were aggregated to generate an Oncoprint^13^ (Table S3). The most common genomic alterations in our clival metastasis cohort were *TP53* (34%, *n* = 20), *PTEN* (31%, *n* = 18), *AR* (29%, *n* = 17), *TMPRSS2–ETS* fusions (22%, *n* = 13), *BRCA2* (15%, *n* = 9), and *ATM* (14%, *n* = 8) (Figure 2).

**Figure 3:**
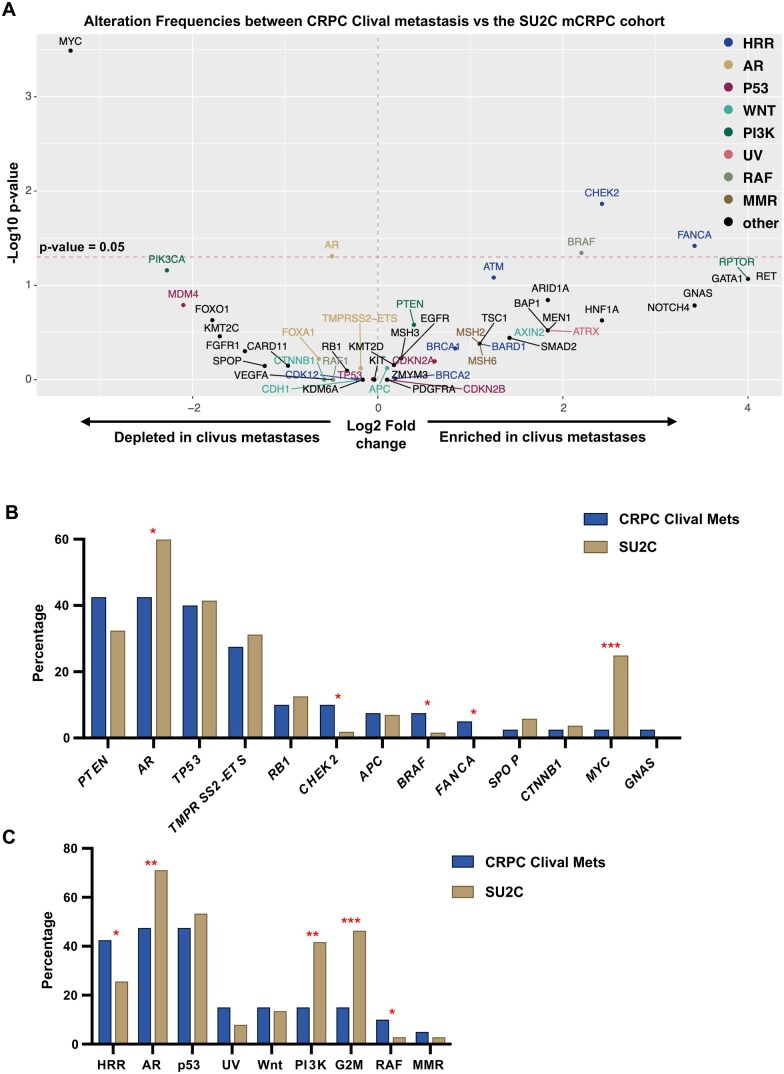
A. Volcano plot comparing alteration frequencies between the mCRPC clival metastasis cohort and the SU2C mCRPC cohort. The x-axis represents the log_2_ fold change in alteration frequency for each gene, and the y-axis shows the −log_10_  *P*-value from the chi-square test. Colors denote pathway classifications. The dashed horizontal line represents a *P*-value of .05. B. Bar graphs depicting the proportion of gene alterations in the mCRPC clival metastases (*n* = 40, blue) compared with the SU2C mCRPC cohort (*n* = 429, gold). C. Bar graphs depicting the proportion of patients in the mCRPC clival metastasis cohort and the SU2C mCRPC cohort with alterations in different pathways. Pathways were defined as follows: HRR (homologous recombination repair: *BRCA1, BRCA2, ATM, PALB2, FANCA, RAD51D, CHEK2, CDK12, BARD1*), AR (AR-related pathway: *AR, TMPRSS2-ETS, FOXA1*), P53 (p53 pathway: *CDKN2A, CDKN2B, TP53, MDM2, MDM4*), WNT (Wnt/β-catenin signaling: *CTNNB1, APC, CDH1, AXIN2*), PI3K (PI3K/AKT/mTOR pathway: *RPTOR, PTEN, PIK3CA, AKT1*), UV (UV damage response: *ATRX, ATM, CHEK1, ERCC2*), G2M (G2/M checkpoint: *TP53, ATM, CHEK2, ATR*), RAF (RAF kinases: *ARAF, BRAF, RAF1*), and MMR (Mismatch repair pathway: *MSH2, MSH6, MLH1, PMS2*). Statistical significance is denoted as ***P <.001, **P <.01, and *P <.05.

To determine whether these alterations were unique to clival involvement, we compared alteration frequencies of patients with mCRPC clival metastases (*n* = 40) (Figure S1) with the publicly available SU2C mCRPC cohort,^11^ which includes 444 metastatic prostate adenocarcinoma samples from 429 patients (Tables S4-S5). Genes significantly enriched in mCRPC clival metastases relative to the SU2C mCRPC cohort included *FANCA* (5% vs 0.5%, *P* = .04), *CHEK2* (10% vs 1.9%, *P* = .01), and *BRAF* (7.5% vs 1.6%, *P* = .045). Conversely, *MYC* (2.5% vs 25%, *P* < .001) and *AR* (42.5% vs 60%, *P* = .049) were significantly depleted in the mCRPC clival metastasis cohort (Figure 3A-B).

We also compared alteration frequencies in the entire clival metastasis cohort (*n* = 59) with the SU2C mCRPC cohort (Figure S2A, B and Table S6). Alterations in *AR, PTEN*, and *TP53* were observed at lower frequencies, as these changes were expected in CRPC*. BRAF* and *CTNNB1* alterations were more frequent in the overall clival metastasis cohort. No other major differences were observed.

The mHSPC clinical metastases (*n* = 19) were also compared with the publicly available mHSPC cohort^12^ (*n* = 424) (Figure S3A, B and Table S7). *PALB2, FGFR2,* and *BRCA2* were the only three genes significantly enriched in the HSPC clival metastasis cohort. Notably, the majority of patients with HSPC clival metastases (14/19, 74%) did not undergo NGS at the time of clival metastasis.

Given that patients may have had sequencing collected at any time during the clinical course, a subgroup analysis was performed that included only patients with sequencing data obtained at the time of clival metastases (*n* = 33). There were no major differences between this subgroup and the overall patient cohort (Figure S4A and B). Although many comparisons did not reach statistical significance, the observed enrichment and depletion patterns suggest potential molecular features that may be unique to clival metastases.

### Homologous recombinant repair (HRR) and RAF kinase pathway alterations

To evaluate whether specific molecular pathways were more or less frequently altered in clival metastases, we grouped genes into common pathways, including the HRR pathway, AR-related signaling, p53 signaling, UV damage response, Wnt/β-catenin signaling, PI3K/AKT/mTOR, G2–M checkpoint, RAF kinases, and mismatch repair (MMR) pathway. We then compared alteration frequencies between the mCRPC clival metastasis cohort and the mCRPC SU2C cohort with respect to these molecular pathways (Figure 3C and Table S8). Notably, the HRR and RAF kinase pathways were significantly enriched in mCRPC clival metastases, demonstrating a 1.7-fold (43% vs 26%, *P* = .035) and 3.6-fold (10% vs 2.8%, *P* = .039) increase in alteration frequency compared to SU2C. Many HRR gene mutations were enriched in the clival cohort, with the exception of *CDK12* (Figure 3A). Genes in the UV damage response pathway also showed approximately double the alteration rate in clival metastases relative to SU2C (15% vs 8%, *P* > .05, Figure 3C). In contrast, the AR-related, PI3K/AKT/mTOR, and G2–M checkpoint pathways exhibited substantially lower alteration frequencies in our clival metastasis cohort, each occurring at approximately half the rate seen in SU2C (0.6-fold, *P* = .004; 0.36-fold, *P* = .002; 0.32-fold, *P* < .001, respectively, Figure 3C). Overall, the enrichment of HRR pathway and UV damage response defects, coupled with depletion of PI3K/AKT/mTOR and G2–M alterations, suggests that clival metastases may harbor unique DNA repair dependencies that could have clinical and therapeutic implications.

## Discussion

Clival metastases are a rare and clinically devastating complication due to their proximity to critical brain structures, and prostate cancer is the most common tumor type giving rise to clivus involvement.^7^^,^^10^ Here, we present the first and largest cohort of patients with prostate cancer with clival metastases and corresponding genetic information, comprising 59 patients and 87 samples with molecular data across six institutions. Clival metastasis was associated with poor prognosis, with a median OS of 15.3 months, and outcomes were worse in those with symptomatic clival disease. Compared with a publicly available metastatic prostate cancer dataset (the SU2C cohort), the clival metastasis cohort was significantly enriched, by approximately 5-fold, for *BRAF* and *CHEK2* alterations and was significantly depleted of *AR* and *MYC* alterations. Correspondingly, alterations in RAF kinase and HRR pathways were markedly enriched, whereas alterations in AR, PI3K/AKT/mTOR, and G2–M checkpoint pathways occurred at roughly half the frequency observed in SU2C. These findings suggest that distinct molecular programs may drive the development of clival metastases in advanced prostate cancer patients.

Clival metastasis was associated with poor clinical features. Most patients presented with advanced disease, with up to 70% diagnosed at de novo stage IV and 55% harboring Gleason grade group 5 histology. Compared with previously reported series, our cohort demonstrated a higher median overall survival (15.3 months vs previously reported 6-15 months^7^^,^^9^^,^^10^), likely because approximately 70% of our patients were asymptomatic at detection, whereas prior reports predominantly included symptomatic patients across diverse primary cancers. Even among patients presenting with cranial nerve palsy, the median OS in our cohort was 13.3 months, although no patient with symptomatic clival involvement in our cohort survived beyond 2 years. Notably, 44% (26/59) of patients were alive at the time of data collection (mostly those without symptomatic clival involvement), indicating that true overall survival may be longer, especially in those where clivus disease may be an incidental finding. Interestingly, none of our patients underwent surgical resection of the clival lesion, reflecting contemporary trends in prostate cancer management and better radiotherapeutic techniques; most surgical resections of clival metastases in the literature occurred before 2015.^14^ Furthermore, increased utilization of whole-body imaging, including molecular PSMA-PET or whole-body MRI imaging, has likely improved the detection of asymptomatic clival metastases, enabling earlier intervention.

Notably, there was a significantly higher frequency of *BRAF* alterations in our clival metastasis cohort (8.5%) compared with metastatic prostate cancer in the SU2C cohort (1.6%) as well as a large second clinical cohort of metastatic prostate cancer (3.3%, *n* = 6210).^15^ A preclinical screening study in prostate cancer cell lines and mouse models suggested that RAF family alterations promoted bone metastases.^16^  *BRAF* is a key activator of the mitogen-activated protein kinase (MAPK) pathway, which promotes cell proliferation. Unlike melanoma and thyroid cancer, non-V600 mutations and *BRAF* rearrangements account for approximately 75% of all *BRAF* alterations in prostate cancer.^15^^,^^17^ Consistent with this pattern, our cohort included three non-V600 missense mutations, one activating gene fusion, and one amplification. Multiple BRAF inhibitors and agents targeting downstream components of the MAPK pathway (eg, MEK inhibitors) are currently under investigation in prostate cancer; however, clinical responses to date have been substantially less impressive than those observed with *BRAF* V600 inhibitors.^18^

Another prominent pathway in the clival metastasis cohort was homologous recombination repair (HRR), with inactivating alterations detected in up to 44% of cases. This rate is substantially higher than that reported in other metastatic prostate cancer cohorts, which range from 20%-30%.^11^^,^^19^^,^^20^ Among HRR-related genes, *BRCA2* was most frequently altered (15.3%), followed by *ATM* (13.6%) and *CHEK2* (8.5%). The frequency of *CHEK2* alterations was notably higher than previously reported rates of approximately 2%,^11^^,^^19^ with most alterations being truncating mutations or deletions. Although prostate cancers harboring HRR gene alterations are often associated with aggressive behavior^21^ and adverse histopathologic features, they may be responsive to poly(ADP-ribose) polymerase (PARP) inhibitors.^20^ In addition to HRR pathway alterations, genes involved in mismatch repair (MMR) and nucleotide excision repair (NER), such as *ERCC2*, were also numerically enriched in clival metastases, suggesting a strong underlying DNA damage–repair deficiency in this cohort.

Our study has several limitations. First, this was a single-arm retrospective cohort study, and including patients from multiple centers introduces clinical heterogeneity and could harbor treatment-selection biases. Although most sequencing assays were performed in Clinical Laboratory Improvement Amendments (CLIA)–certified laboratories, technical variability across platforms in terms of gene coverage and detectable alteration types is likely, contributing to data heterogeneity. The SU2C cohort was sequenced in a research format that utilized different normalization methods and may not be a directly comparable assay. The incomplete clinical metadata within the SU2C dataset, specifically the absence of initial staging and metastatic sites, precludes a matched comparison of potential confounding factors. Additionally, the higher prevalence of neuroendocrine histology in the SU2C cohort compared to our clival cohort (10% vs 3%) may introduce inherent bias in survival outcomes. While 56% (33/59) of patients underwent sequencing at the time of clival metastasis, 64% (21/33) of these samples were blood-based ctDNA next-generation sequencing (NGS), which has lower sensitivity than tissue-based NGS, especially for detecting amplifications and deletions. Consequently, molecular alterations acquired specifically within clival metastases may have been underappreciated. In addition, none of the patients underwent NGS testing from the clivus metastases, so the genomic landscape may have been slightly different in the clivus lesions themselves. Due to the exploratory nature of this study and the rarity of the clinical entity, p-values were not adjusted for multiple comparisons to avoid omitting potentially significant biological associations. The enrichment and depletion analyses of genes and pathways in this study are associative and should be interpreted as hypothesis-generating rather than causal evidence of clival metastatic tropism. Furthermore, because this study focused on DNA alterations and did not include RNA expression data, our ability to infer pathway-level functional activity in this patient cohort was limited. Finally, it is possible that clivus metastases are simply a manifestation of very high osseous burden (or high metastatic burden overall), rather than representing a specific proclivity to clival spread, and thus the underlying genomic mechanisms may not be directly related to biological tropism towards the clivus bone.

## Conclusion

In conclusion, clival metastasis represents a rare but highly aggressive manifestation of advanced prostate cancer, associated with poor survival. In this largest multi-institutional cohort to date, we demonstrate that prostate cancer clival metastases harbor a distinct molecular landscape characterized by enrichment of DNA damage–repair alterations (particularly within the HRR pathway) and RAF kinase pathway alterations, alongside relative depletion of AR-related and PI3K/AKT/mTOR pathway alterations. These findings suggest that clival metastases may arise through unique biological mechanisms and may exhibit distinct therapeutic vulnerabilities. Although limited by retrospective design and molecular heterogeneity, our study provides the first comprehensive genomic characterization of prostate cancers giving rise to clival metastases and highlights the need for heightened clinical awareness, early skull-base imaging, and consideration of targeted therapeutic strategies, particularly HRR–directed approaches, in this high-risk population.

## Supplementary Material

oyag074_Supplementary_Data

## Data Availability

Individual-level clinical and genomic data are not publicly available due to institutional restrictions and patient privacy considerations. Aggregate genomic alteration frequencies and summary-level data generated during this study are included in the article and its supplementary materials.

## References

[1] Bray F , Laversanne M, Sung H, et al Global cancer statistics 2022: GLOBOCAN estimates of incidence and mortality worldwide for 36 cancers in 185 countries. CA Cancer J Clin. 2024;74:229-263. 10.3322/caac.21834 38572751

[2] Siegel RL , Giaquinto AN, Jemal A. Cancer statistics, 2024. CA Cancer J Clin. 2024;74:12-49. 10.3322/caac.21820 38230766

[3] American Cancer Society. Cancer Facts & Figures 2025. Atlanta: American Cancer Society, 2025.

[4] Surveillance, Epidemiology, and End Results (SEER) Program (www.seer.cancer.gov). SEER*Stat Database: Mortality—All COD, Aggregated with State, Total U.S. (1969-2023). 2025. https://seer.cancer.gov/statfacts/, accessed on 1 Dec. 2025.

[5] Gandaglia G , Abdollah F, Schiffmann J, et al Distribution of metastatic sites in patients with prostate cancer: a population-based analysis. Prostate. 2014;74:210-216. 10.1002/pros.22742 24132735

[6] Sabbatini P , Larson SM, Kremer A, et al Prognostic significance of extent of disease in bone in patients with androgen-independent prostate cancer. J Clin Oncol. 1999;17:948-957. 10.1200/JCO.1999.17.3.948 10071289

[7] Carretta A , Sollini G, Guaraldi F, et al Clival metastases: single-center retrospective case series and literature review. J Clin Med. 2024;13:2580. 10.3390/jcm13092580 38731109 PMC11084723

[8] Sturgis R , Mack A, Kim S, et al Symptom outcomes of cancer patients with clival metastases treated with radiotherapy: a study of 44 patients. Anticancer Res. 2021;41:5001-5006. 10.21873/anticanres.15314 34593448

[9] Jozsa F , Das JM. Metastatic lesions of the clivus: a systematic review. World Neurosurg. 2022;158:190-204. 10.1016/j.wneu.2021.11.105 34861450

[10] Huq S , Shanahan RM, Adida S, et al Gamma knife radiosurgery for clival metastasis: case series and systematic review. J Neurooncol. 2024;168:171-183. 10.1007/s11060-024-04648-9 38598088

[11] Abida W , Cyrta J, Heller G, et al Genomic correlates of clinical outcome in advanced prostate cancer. Proc Natl Acad Sci USA. 2019;116:11428-11436. 10.1073/pnas.1902651116 31061129 PMC6561293

[12] Stopsack KH , Nandakumar S, Wibmer AG, et al Oncogenic genomic alterations, clinical phenotypes, and outcomes in metastatic Castration-Sensitive prostate cancer. Clin Cancer Res. 2020;26:3230-3238. 10.1158/1078-0432.CCR-20-0168 32220891 PMC7334067

[13] Gu Z , Eils R, Schlesner M. Complex heatmaps reveal patterns and correlations in multidimensional genomic data. Bioinformatics. 2016;32:2847-2849. 10.1093/bioinformatics/btw313 27207943

[14] Zacharia BE , Romero FR, Rapoport SK, et al Endoscopic endonasal management of metastatic lesions of the anterior skull base: case series and literature review. World Neurosurg. 2015;84:1267-1277. 10.1016/j.wneu.2015.05.061 26079759

[15] Chehrazi-Raffle A , Tukachinsky H, Toye E, et al Unique spectrum of activating BRAF alterations in prostate cancer. Clin Cancer Res. 2023;29:3948-3957. 10.1158/1078-0432.CCR-23-1393 37477913 PMC10543965

[16] Faltermeier CM , Drake JM, Clark PM, et al Functional screen identifies kinases driving prostate cancer visceral and bone metastasis. Proc Natl Acad Sci USA. 2016;113:E172-E181. 10.1073/pnas.1521674112 26621741 PMC4720329

[17] Owsley J , Stein MK, Porter J, et al Prevalence of class I–III BRAF mutations among 114,662 cancer patients in a large genomic database. Exp Biol Med (Maywood). 2021;246:31-39. 10.1177/1535370220959657 33019809 PMC7797994

[18] Toye E , Chehrazi-Raffle A, Hwang J, et al Targeting the multifaceted BRAF in cancer: new directions. Oncotarget. 2024;15:486-492. 10.18632/oncotarget.28612 39018217 PMC11254297

[19] Olmos D , Lorente D, Alameda D, et al Treatment patterns and outcomes in metastatic castration-resistant prostate cancer patients with and without somatic or germline alterations in homologous recombination repair genes. Ann Oncol. 2024;35:458-472. 10.1016/j.annonc.2024.01.011 38417742

[20] Bono J de MJ , Fizazi K, et al Olaparib for metastatic Castration-Resistant prostate cancer. N Engl J Med. 2020;382:2091-2102. 10.1056/NEJMoa1911440 32343890

[21] Pan J , Zhu B, Wu J, et al Homologous recombination repair gene alteration is strongly associated with more prostate-specific membrane antigen-positive metastases in newly diagnosed hormone-sensitive prostate cancer with ≤5 conventional imaging defined distant metastases. Eur J Nucl Med Mol Imaging. 2025;52:4054-4064. 10.1007/s00259-025-07278-z 40237795

