# Supplementary material for: Molecular landscape of prostate cancers with clival metastases
Source: Oncologist. 2026 Mar 4;31(4):oyag074. doi: 10.1093/oncolo/oyag074 (PMC12995431; doi:10.1093/oncolo/oyag074)
Supplement: oyag074_Supplementary_Data [file oyag074_supplementary_data.zip › SupplementaryTable1-8.pdf]

Supplementary Tables

Supplementary Table 1. Details of NGS sequencing

Supplementary Table 2. Sample-level data of all mutations

Supplementary Table 3. Patient-level data of all mutations

Supplementary Table 4. Demographic data of patients in the mCRPC SU2C cohort and patients with clival metastasis

Supplementary Table 5. Statistics for a comparison between the mCRPC clival metastatic cohort and the SU2C cohort

Supplementary Table 6. Statistics for a comparison between the overall clival metastatic cohort and the SU2C cohort

Supplementary Table 7. Statistics for a comparison between the mHSPC clival metastatic cohort and the Stopsack mHSPC cohort

Supplementary Table 8. Statistics for pathway analysis

Supplementary Table 1. Details of NGS sequencing

	**Number of assays N = 87**	**%**	**Number of patients N = 59**	**%**
**Sequencing platform**				
Tissue-based NGS	42	48.3%	39	66.1%
Blood-based NGS	45	51.7%	36	61.0%
**Timing of sample collection**				
Sample collected at diagnosis	32	36.8%	31	52.5%
Sample collected at M1	27	31.0%	26	44.1%
Sample collected at or after clival metastases	43	49.4%	33	55.9%

Supplementary Table 2

	UMN_01	UMN_02	UMN_03.1	UMN_03.2	UMN_04.1	UMN_04.2	UMN_05	UMN_06.1	UMN_06.2	UMN_07.1
TMPRSS2-ETS	FUSION									
PTEN	T319fs*1	165-2A>C splice site				D52H				
FOXA1				R262_Q263 insHLRR						
KMT2C			P1879fs	P1879fs						
AR				L702H			T878A;H875Y			
FAT1		3266-4_3301del40 splice site								
TP53	I254S									
ATM					R250*;DEL	S276L				
SMAD2			S276L	S276L						
EGFR							A583T			
BAP1		N413fs*4								
BRAF							AMP			
KIT							AMP			
PDGFRA							AMP			
MEN1				Q7*						
APC										
CHEK2										
ERCC3										
IKZF1										
LRP1B										
RB1										
KDM6A										
ZMYM3										
VEGFA										
ARID1A										DEL
MYC										
KMT2D										
PIK3CA										
CDKN2A										
SMAD4										
PALB2										
FOXO1										
BRCA1										
MSH6										
BRCA2										
CTNNB1										
ERCC2										
SMARCA2										
CDK12										
FANCA										
SMARCA4										
SPOP										
GATA1										
FGFR1										
CDH1										
BARD1										
RAF1										
RET										
RPTOR										
MLH1										
GNAS										
NOTCH4										
HNF1A										
FGFR2										
CDKN2B										
ATRX										
MDM4										
AXIN2										
MSH2										
MSH3										
CARD11										
TSC1										

Supplementary Table 2

UMN_07.2	UMN_08.1	UMN_08.2	UMN_09	Mayo_01.1	Mayo_01.2	Mayo_02.1	Mayo_02.2	Mayo_03.1	Mayo_03.2	Mayo_04.1	Mayo_04.2
				DEL	DEL	R308fs*		Y68*	Y68*		
		AMP									
R280K						L265P	L265P				
	c.1235+3del	L612R									
		V769M									
		R682W									
			S1465fs*								
									Y424H		
			AMP								
	Q827fs						S492fs				
		AMP									
			S1982fs*								
			AMP								

Supplementary Table 2

Mayo_04.3	Mayo_04.4	Mayo_04.5	Mayo_04.6	Mayo_04.7	Mayo_05	Mayo_06	Mayo_07	Mayo_08.1	Mayo_08.2	Mayo_09	Mayo_10
										FUSION	
										DEL	
						AMP		T878A	T878A	AMP	
				A138V			R156P				
			R1882*	R1882*							
							V528F			BRAF-EXOC4 fusion	
				E1540*							
G165D	V234fs		G165D	G165D							
						DEL					
										C1410*	
										W575fs	
										AMP	
						AMP					

Supplementary Table 2

Mayo_11.1	Mayo_11.2	Mayo_12	Utah_01	Utah_02.1	Utah_02.2	Utah_03	Utah_04.1	Utah_04.2	Utah_05	Utah_06	Utah_07
FUSION								FUSION		FUSION	FUSION
DEL	DEL	DEL						N329fs		DEL	
		AMP							W742C; W742L; T878A		
									R248Q	R175H	
					C718fs	Y1544fs*1					
									DEL		
								DEL		DEL	
										E545K	
			R170fs								
								DEL			
									DEL		
									K923fs		
											F2130fs
											S37F
			c.594-12 (splice site)								
			DEL								

Supplementary Table 2

WCM_01	WCM_02	WCM_03.1	WCM_03.2	WCM_04	WCM_05	WCM_06	FHCC_01	FHCC_02	FHCC_03	FHCC_04.1	FHCC_04.2
		FUSION									FUSION
	DEL									DEL	DEL
											H832Tfs*7
	AMP					H875Y;L702H					AMP
	N268Efs*4				splice site; A161T;R213*						
										DEL	
		F1515Lfs*8	F1515Lfs*8			Multiple truncating alterations					
									E630fs*61		
		3706_3715+123del133 splice site									
					L291Ffs*7						
		K2162Nfs*5	K2162Nfs*5					inversion exon 10-18			
					S37F						
E454fs*1											
1776+1G>T splice site											
				splice site							
						F102C					
		Rearrangement intron 8									

Supplementary Table 2

FHCC_05.1	FHCC_05.2	FHCC_05.3	FHCC_05.4	FHCC_06	FHCC_07	FHCC_08	FHCC_09	FHCC_10	FHCC_11	FHCC_12	FHCC_13
FUSION		FUSION		FUSION							FUSION
DEL					DEL	T319Nfs*6		DEL			
				AMP	AMP			DEL exons 5-7; FU rearrangement intron 4; AMP			
G244D			G244D; F13	I254S		F134C		R175H			R158H
				R447*						C2624fs	
									G469S		
									T367fs*15		T3g7Mfs*15
	DEL	DEL									DEL
							S33C				
					GATA1-SCFD2 rearrangement						
					AMP						
								687+2T>A splice site			
								V636fs*1			
								rearrangment intron 6			
								RET-NAA16			

Supplementary Table 2

FHCC_14	FHCC_15	FHCC_16.1	FHCC_16.2	FHCC_17	FHCC_18	FHCC_19.1	FHCC_19.2	FHCC_20	FHCC_21.1	FHCC_21.2	ICR_01
		FUSION							FUSION		
						E253fs*6					
		DEL								AMP	
										AMP	
R110P						V274L			M237I;DEL	M237I	
			C176G	P278S	P177R	R1882*					
										DEL	
									AMP		
											DEL
DEL	DEL										DEL
						S45C					
		AMP									
								exon 10-11 rearrangement			
										R265C	
										NOTCH4-NUDT3 fusion	
										AMP	
											DEL

Supplementary Table 2

ICR_02	ICR_03.1	ICR_03.2	ICR_04.1	ICR_04.2
	AMP		L702H; H87	L702H; H875Y
	DEL			
				R81Q;G67S
DEL				
				R917*
[illegible]				
AMP				
	AMP			
			E405Gfs*56	
			R621*	
				K383fs*32
				R888C

Supplementary Table 3

	UMN_01	UMN_02	UMN_03	UMN_04	UMN_05	UMN_06	UMN_07	UMN_08
**no. of samples**	1	1	2	2	1	2	2	2
**Tissue (T-tissue, L-liquid(blood), B-both)**	T	T	B	B	L	B	B	B
**Patho - Adenocarcinoma (A), Neuroendocrine (N)**	A	A	A	A	A	A	A	A
TMPRSS2-ETS	FU							
MYB-NFIB								
PTEN	TR	SP		MS				
FOXA1			INF					
KMT2C			TR					
AR			MS		MS			AMP
FAT1		SP						
TP53	MS						MS	
ATM				TR;DEL				MS;SP
SMAD2			MS					
EGFR					MS			MS
BAP1		TR						
BRAF					AMP			MS
KIT					AMP			
PDGFRA					AMP			
MEN1			TR					
APC								
CHEK2								
ERCC3								
IKZF1								
LRP1B								
RB1								
KDM6A								
ZMYM3								
VEGFA								
ARID1A							DEL	
MYC								
KMT2D								TR
PIK3CA								AMP
CDKN2A								
SMAD4								
PALB2								
FOXO1								
BRCA1								
MSH6								
BRCA2								
CTNNB1								
ERCC2								
SMARCA2								
CDK12								
FANCA								
SMARCA4								
SPOP								
GATA1								
FGFR1								
CDH1								
BARD1								
RAF1								
RET								
RPTOR								
MLH1								
GNAS								
NOTCH4								
HNF1A								
FGFR2								
CDKN2B								
ATRX								
MDM4								
AXIN2								
MSH2								
MSH3								
CARD11								
TSC1								

Supplementary Table 3

UMN_09	Mayo_01	Mayo_02	Mayo_03	Mayo_04	Mayo_05	Mayo_06	Mayo_07	Mayo_08	Mayo_09	Mayo_10	Mayo_11	Mayo_12	Utah_01
1	2	2	2	7	1	1	1	2	1	1	2	1	1
T	T	B	L	B	T	T	L	L	T	T	B	L	T
A	N	A	A	A	A	A	A	A	A	A	N	A	A
									FU		FU		
	DEL	TR	TR						DEL		DEL	DEL	
						AMP		MS	AMP			AMP	
		MS		MS			MS						
				TR									
							MS		FU;SP				
TR				TR									
			MS	MS;TR									
						DEL							
									TR				
									TR				
									AMP				
AMP						AMP							
		TR											
													TR
TR													
													SP
													DEL
AMP													

Supplementary Table 3

Utah_02	Utah_03	Utah_04	Utah_05	Utah_06	Utah_07	WCM_01	WCM_02	WCM_03	WCM_04	WCM_05	WCM_06	FHCC_01	FHCC_02
2	1	2	1	1	1	1	1	2	1	1	1	1	1
B	T	B	L	T	T	L	L	L	L	L	L	T	T
A	A	A	A	A	A	A	A	A	A	A	A	A	A
		FU		FU	FU			FU					
		TR		DEL			DEL						
			MS				AMP				MS		
			MS	MS			TR			TR;MS;SP			
TR	TR												
								TR			TR		
			DEL										
		DEL		DEL									
								SP					
				MS									
		DEL											
			DEL							TR			
			TR										
					TR			TR					FU
					MS					MS			
						TR							
						SP							
									SP				
											MS		
								FU					

Supplementary Table 3

FHCC_03	FHCC_04	FHCC_05	FHCC_06	FHCC_07	FHCC_08	FHCC_09	FHCC_10	FHCC_11	FHCC_12	FHCC_13	FHCC_14	FHCC_15	FHCC_16
1	2	4	1	1	1	1	1	1	1	1	1	1	2
T	B	L	L	L	B	T	L	T	T	L	L	T	B
A	A	A	A	A	A	A	A	A	A	A	A	A	A
	FU	FU	FU							FU			FU
	DEL	DEL		DEL	TR		DEL						DEL
	TR												
	AMP		AMP	AMP			DEL;AMP;FU						
		MS	MS		MS		MS			MS	MS		MS
			TR						TR				
	DEL												
								MS					
								TR		TR			
TR													
		DEL								DEL	DEL	DEL	
						MS							
				FU									
				AMP									
							SP						
							TR						
							FU						
							FU						
													AMP

Supplementary Table 3

FHCC_17	FHCC_18	FHCC_19	FHCC_20	FHCC_21	ICR_01	ICR_02	ICR_03	ICR_04
1	1	2	1	2	1	1	2	2
L	L	B	T	T	T	T	B	B
A	A	A	A	A	A	A	A	A
				FU				
		TR						
				AMP				
				AMP			AMP	MS
MS	MS	MS		MS;DEL				
		TR						
				DEL			DEL	
					DEL			MS
					DEL			
		MS						
						DEL		
								TR
			FU					
				MS				
				FU				
				AMP				
					DEL			
						AMP		
							AMP	
								TR
								TR
								TR
								MS

Supplementary Table 4. Demographic data of patients in the mCRPC SU2C cohort and patients with clival metastasis

	**SU2C cohort**		**Clival metastatic cohort**	
	**Median (range)**		**Median (range)**	
**Age**				
Age at diagnosis (years)	61.2 (38.6, 89.0)		63.1 (43.0, 85.4)	
Age at procurement (years)	67.3 (39.5, 95.0)		69.8 (52.9, 88.6)	
**Tissue types**	**N = 429**	**%**	**N=59**	**%**
HSPC	0	0%	19	32.2%
CRPC	429	100%	40	67.8%
**Pathology**				
Adenocarcinoma	319	74.4%	57	96.6%
Neuroendocrine	42	9.8%	2	3.4%
NA	68	15.9%	0	0%
**Gleason score**				
6	27	6.3%	0	0%
7	103	24.0%	14	23.7%
8	67	15.6%	0	0%
9	123	28.7%	30	50.8%
10	24	5.6%	3	5.1%
NA	84	19.6%	12	20.3%
**Tissue sites**	**N=444**	**%**	**N=87**	**%**
Lymph nodes	167	37.6%	8	9.2%
Bone	160	36.0%	10	11.5%
Liver	64	14.4%	3	3.4%
Other Soft tissue	29	6.5%	1	1.1%
Prostate	12	2.7%	19	21.8%
Lung	7	1.6%	1	1.1%
Other	3	0.7%	0	0%
Blood	0	0%	45	51.7%
NA	2	0.5%	0	0%

Supplementary Table 5

Gene	sum_1	length_1	freq1	sum_2	length_2	freq2	Ratio clival/SU	statistic/estim
PTEN	17	40	42.5	139	429	32.4009324	1.311690647	1.256898239
AR	17	40	42.5	257	429	59.90675991	0.709435798	3.875475898
TP53	16	40	40	178	429	41.49184149	0.964044944	0.000236809
TMPRSS2-ETS	11	40	27.5	134	429	31.23543124	0.880410448	0.096131041
ATM	6	40	15	27	429	6.293706294	2.383333333	3.013363155
BRCA2	5	40	12.5	47	429	10.95571096	1.140957447	0.001172508
CHEK2	4	40	10	8	429	1.864801865	5.3625	5.805678454
RB1	4	40	10	54	429	12.58741259	0.794444444	0.77199947
BRAF	3	40	7.5	7	429	1.631701632	4.596428571	4.858407494
APC	3	40	7.5	30	429	6.993006993	1.0725	1.078225304
FOXA1	3	40	7.5	50	429	11.65501166	0.6435	0.615142079
FANCA	2	40	5	2	429	0.466200466	10.725	11.10695312
ARID1A	2	40	5	6	429	1.398601399	3.575	3.693322695
CDKN2A	2	40	5	14	429	3.263403263	1.532142857	1.558383755
EGFR	2	40	5	19	429	4.428904429	1.128947368	1.135394029
KMT2D	2	40	5	19	429	4.428904429	1.128947368	1.135394029
CDK12	2	40	5	25	429	5.827505828	0.858	0.85083109
GATA1	1	40	2.5	0	429	0	#DIV/0!	#NUM!
RET	1	40	2.5	0	429	0	#DIV/0!	#NUM!
RPTOR	1	40	2.5	0	429	0	#DIV/0!	#NUM!
GNAS	1	40	2.5	1	429	0.233100233	10.725	10.84920623
NOTCH4	1	40	2.5	1	429	0.233100233	10.725	10.84920623
HNF1A	1	40	2.5	2	429	0.466200466	5.3625	5.436698187
BAP1	1	40	2.5	3	429	0.699300699	3.575	3.624452309
MEN1	1	40	2.5	3	429	0.699300699	3.575	3.624452309
ATRX	1	40	2.5	3	429	0.699300699	3.575	3.624452309
SMAD2	1	40	2.5	4	429	0.932400932	2.68125	2.715692104
AXIN2	1	40	2.5	4	429	0.932400932	2.68125	2.715692104
MSH6	1	40	2.5	5	429	1.165501166	2.145	2.169546549
BARD1	1	40	2.5	5	429	1.165501166	2.145	2.169546549
MSH2	1	40	2.5	5	429	1.165501166	2.145	2.169546549
TSC1	1	40	2.5	5	429	1.165501166	2.145	2.169546549
BRCA1	1	40	2.5	6	429	1.398601399	1.7875	1.804801761
MSH3	1	40	2.5	9	429	2.097902098	1.191666667	1.196088678
PDGFRA	1	40	2.5	10	429	2.331002331	1.0725	1.074218663
ZMYM3	1	40	2.5	10	429	2.331002331	1.0725	1.074218663
CDKN2B	1	40	2.5	10	429	2.331002331	1.0725	1.074218663
KIT	1	40	2.5	11	429	2.564102564	0.975	0.974411758
KDM6A	1	40	2.5	12	429	2.797202797	0.89375	0.891224843
CDH1	1	40	2.5	12	429	2.797202797	0.89375	0.891224843
VEGFA	1	40	2.5	15	429	3.496503497	0.715	0.708151866
RAF1	1	40	2.5	15	429	3.496503497	0.715	0.708151866
CTNNB1	1	40	2.5	16	429	3.72960373	0.6703125	0.662356137
CARD11	1	40	2.5	21	429	4.895104895	0.510714286	0.498738731
SPOP	1	40	2.5	25	429	5.827505828	0.429	0.414920794
FGFR1	1	40	2.5	29	429	6.75990676	0.369827586	0.354206608
KMT2C	1	40	2.5	35	429	8.158508159	0.306428571	0.289137384
FOXO1	1	40	2.5	37	429	8.624708625	0.289864865	0.272134155
MDM4	1	40	2.5	46	429	10.72261072	0.233152174	0.213906539
PIK3CA	1	40	2.5	52	429	12.12121212	0.20625	0.186280261
MYC	1	40	2.5	107	429	24.94172494	0.100233645	0.077375309

*adjusted log2FC

Supplementary Table 5

p.value	method	conf.low	conf.high	Log2_FC	Log10_p
0.262239029	Pearson's Chi-squared test with Yates' continu			0.391427511	0.581302672
0.048996417	Pearson's Chi-squared test with Yates' continu			-0.495255965	1.309835674
0.987722161	Pearson's Chi-squared test with Yates' continu			-0.052827688	0.005365202
0.756522886	Pearson's Chi-squared test with Yates' continu			-0.183751829	0.121177929
0.082580787	Pearson's Chi-squared test with Yates' continu			1.252980741	1.083120982
0.972684251	Pearson's Chi-squared test with Yates' continu			0.190244986	0.012028116
0.013656546	Fisher's Exact	1.219977648	22.97072902	2.422905743	1.864659138
0.803938043	Fisher's Exact	0.192079867	2.280608305	-0.33198176	0.094777419
0.045316555	Fisher's Exact	0.778698698	22.40774461	2.200513321	1.343743112
0.753650073	Fisher's Exact	0.201073938	3.73157811	0.100977648	0.122830254
0.602791334	Fisher's Exact	0.117092535	2.057781186	-0.635987946	0.219833
0.038155072	Fisher's Exact	0.784921531	157.1231283	3.422905743	1.418447722
0.143115669	Fisher's Exact	0.353044823	21.59056683	1.837943242	0.844312816
0.63765299	Fisher's Exact	0.165887063	7.190792325	0.615550821	0.195415599
0.69743043	Fisher's Exact	0.12364695	5.006148859	0.174978229	0.156499107
0.69743043	Fisher's Exact	0.12364695	5.006148859	0.174978229	0.156499107
1	Fisher's Exact	0.094128683	3.639123018	-0.220950447	0
0.085287846	Fisher's Exact	0.274999981	#NUM!	4*	1.069112851
0.085287846	Fisher's Exact	0.274999981	#NUM!	4*	1.069112851
0.085287846	Fisher's Exact	0.274999981	#NUM!	4*	1.069112851
0.163468372	Fisher's Exact	0.136334602	856.6723676	3.422905743	0.786566261
0.163468372	Fisher's Exact	0.136334602	856.6723676	3.422905743	0.786566261
0.235119904	Fisher's Exact	0.090491145	106.642456	2.422905743	0.628710605
0.300774848	Fisher's Exact	0.067639893	46.35150801	1.837943242	0.521758485
0.300774848	Fisher's Exact	0.067639893	46.35150801	1.837943242	0.521758485
0.300774848	Fisher's Exact	0.067639893	46.35150801	1.837943242	0.521758485
0.360923248	Fisher's Exact	0.053951636	28.33925138	1.422905743	0.442585143
0.360923248	Fisher's Exact	0.053951636	28.33925138	1.422905743	0.442585143
0.416016071	Fisher's Exact	0.044835489	20.09875814	1.100977648	0.380889892
0.416016071	Fisher's Exact	0.044835489	20.09875814	1.100977648	0.380889892
0.416016071	Fisher's Exact	0.044835489	20.09875814	1.100977648	0.380889892
0.416016071	Fisher's Exact	0.044835489	20.09875814	1.100977648	0.380889892
0.466468247	Fisher's Exact	0.038328534	15.46926682	0.837943242	0.331177914
0.593647088	Fisher's Exact	0.026625621	9.03384167	0.252980741	0.226471658
1	Fisher's Exact	0.024144771	7.914003592	0.100977648	0
1	Fisher's Exact	0.024144771	7.914003592	0.100977648	0
1	Fisher's Exact	0.024144771	7.914003592	0.100977648	0
1	Fisher's Exact	0.02207782	7.036037673	-0.036525876	0
1	Fisher's Exact	0.020329161	6.328278985	-0.162056758	0
1	Fisher's Exact	0.020329161	6.328278985	-0.162056758	0
1	Fisher's Exact	0.016395685	4.847595524	-0.483984853	0
1	Fisher's Exact	0.016395685	4.847595524	-0.483984853	0
1	Fisher's Exact	0.015396387	4.493264932	-0.577094257	0
0.710262622	Fisher's Exact	0.011752037	3.27628982	-0.96941168	0.14858104
0.714639105	Fisher's Exact	0.009846273	2.681569334	-1.220950447	0.145913223
0.498432799	Fisher's Exact	0.008448944	2.262466387	-1.435075253	0.302393387
0.346716453	Fisher's Exact	0.0069354	1.823878808	-1.706377274	0.460025549
0.234045472	Fisher's Exact	0.00653714	1.710998437	-1.786547623	0.630699757
0.161923863	Fisher's Exact	0.005164549	1.330132008	-2.100656213	0.790689144
0.06928758	Fisher's Exact	0.004508543	1.152323473	-2.277533976	1.159344609
0.000324587	Fisher's Exact	0.001892141	0.46923602	-3.318561244	3.48866907

Supplementary Table 6

Gene	sum_1	length_1	freq1	sum_2	length_2	freq2	Ratio clival/SL	statistic/estim
TP53	20	59	33.89830508	178	429	41.49184149	0.816987241	0.945434058
PTEN	18	59	30.50847458	139	429	32.4009324	0.941592489	0.020488898
AR	17	59	28.81355932	257	429	59.90675991	0.480973422	19.12228488
TMPRSS2-ETS	13	59	22.03389831	134	429	31.23543124	0.70541361	1.672056402
BRCA2	9	59	15.25423729	47	429	10.95571096	1.39235485	0.5677062
ATM	8	59	13.55932203	27	429	6.293706294	2.154425612	3.093610596
BRAF	5	59	8.474576271	7	429	1.631701632	5.1937046	5.548776646
CHEK2	5	59	8.474576271	8	429	1.864801865	4.544491525	4.846915083
RB1	5	59	8.474576271	54	429	12.58741259	0.673258004	0.643523273
CTNNB1	4	59	6.779661017	16	429	3.72960373	1.81779661	1.874178589
APC	4	59	6.779661017	30	429	6.993006993	0.969491525	0.967333462
FOXA1	3	59	5.084745763	50	429	11.65501166	0.436271186	0.406630691
FANCA	2	59	3.389830508	2	429	0.466200466	7.271186441	7.435599306
BAP1	2	59	3.389830508	3	429	0.699300699	4.847457627	4.955320027
ARID1A	2	59	3.389830508	6	429	1.398601399	2.423728814	2.467340276
BRCA1	2	59	3.389830508	6	429	1.398601399	2.423728814	2.467340276
KDM6A	2	59	3.389830508	12	429	2.797202797	1.211864407	1.218760653
CDKN2A	2	59	3.389830508	14	429	3.263403263	1.03874092	1.040022627
EGFR	2	59	3.389830508	19	429	4.428904429	0.765388046	0.757548252
KMT2D	2	59	3.389830508	19	429	4.428904429	0.765388046	0.757548252
CDK12	2	59	3.389830508	25	429	5.827505828	0.581694915	0.567562553
PIK3CA	2	59	3.389830508	52	429	12.12121212	0.279661017	0.254849548
MYC	2	59	3.389830508	107	429	24.94172494	0.135910027	0.105865323
RET	1	59	1.694915254	0	429	0	#DIV/0!	#NUM!
RPTOR	1	59	1.694915254	0	429	0	#DIV/0!	#NUM!
GATA1	1	59	1.694915254	0	429	0	#DIV/0!	#NUM!
GNAS	1	59	1.694915254	1	429	0.233100233	7.271186441	7.326614638
NOTCH4	1	59	1.694915254	1	429	0.233100233	7.271186441	7.326614638
MLH1	1	59	1.694915254	2	429	0.466200466	3.63559322	3.665874305
HNF1A	1	59	1.694915254	2	429	0.466200466	3.63559322	3.665874305
FGFR2	1	59	1.694915254	2	429	0.466200466	3.63559322	3.665874305
MEN1	1	59	1.694915254	3	429	0.699300699	2.423728814	2.442091679
PALB2	1	59	1.694915254	3	429	0.699300699	2.423728814	2.442091679
ATRX	1	59	1.694915254	3	429	0.699300699	2.423728814	2.442091679
FAT1	1	59	1.694915254	4	429	0.932400932	1.81779661	1.829068134
SMAD2	1	59	1.694915254	4	429	0.932400932	1.81779661	1.829068134
ERCC2	1	59	1.694915254	4	429	0.932400932	1.81779661	1.829068134
AXIN2	1	59	1.694915254	4	429	0.932400932	1.81779661	1.829068134
MSH6	1	59	1.694915254	5	429	1.165501166	1.454237288	1.460756788
BARD1	1	59	1.694915254	5	429	1.165501166	1.454237288	1.460756788
MSH2	1	59	1.694915254	5	429	1.165501166	1.454237288	1.460756788
TSC1	1	59	1.694915254	5	429	1.165501166	1.454237288	1.460756788
SMARCA4	1	59	1.694915254	6	429	1.398601399	1.211864407	1.214993824
MSH3	1	59	1.694915254	9	429	2.097902098	0.807909605	0.804928961
PDGFRA	1	59	1.694915254	10	429	2.331002331	0.727118644	0.722841729
ZMYM3	1	59	1.694915254	10	429	2.331002331	0.727118644	0.722841729
CDKN2B	1	59	1.694915254	10	429	2.331002331	0.727118644	0.722841729
KIT	1	59	1.694915254	11	429	2.564102564	0.661016949	0.655659853
CDH1	1	59	1.694915254	12	429	2.797202797	0.605932203	0.599661121
VEGFA	1	59	1.694915254	15	429	3.496503497	0.484745763	0.476418168
RAF1	1	59	1.694915254	15	429	3.496503497	0.484745763	0.476418168
SMARCA2	1	59	1.694915254	17	429	3.962703963	0.427716849	0.418396181
CARD11	1	59	1.694915254	21	429	4.895104895	0.346246973	0.335486053
SPOP	1	59	1.694915254	25	429	5.827505828	0.290847458	0.279089478
FGFR1	1	59	1.694915254	29	429	6.75990676	0.250730567	0.238241421
KMT2C	1	59	1.694915254	35	429	8.158508159	0.207748184	0.194466946
FOXO1	1	59	1.694915254	37	429	8.624708625	0.196518552	0.18302887
MDM4	1	59	1.694915254	46	429	10.72261072	0.15806927	0.14386112

**adjusted Log2FC*

Supplementary Table 6

p.value	method	conf.low	conf.high	FC	Log2_FC	Log10_p
0.330884248	Pearson's Chi-squared test with Yates' continu			0.816987241	-0.291614548	0.480323907
0.886180061	Pearson's Chi-squared test with Yates' continu			0.941592489	-0.086825283	0.052478026
1.22605E-05	Pearson's Chi-squared test with Yates' continu			0.480973422	-1.05597092	4.911492044
0.195983323	Pearson's Chi-squared test with Yates' continu			0.70541361	-0.503458684	0.707780884
0.451171854	Pearson's Chi-squared test with Yates' continu			1.39235485	0.477526938	0.345658002
0.078600224	Pearson's Chi-squared test with Yates' continu			2.154425612	1.107303286	1.104576219
0.008875566	Fisher's Exact	1.339831521	21.13092088	5.1937046	2.376763961	2.051803959
0.013092682	Fisher's Exact	1.202866356	17.5179377	4.544491525	2.184118883	1.882971394
0.521811849	Fisher's Exact	0.192366052	1.700042636	0.673258004	-0.570768619	0.282486063
0.285229525	Fisher's Exact	0.440079128	6.09898522	1.81779661	0.862190788	0.544805521
1	Fisher's Exact	0.23855867	2.899128822	0.969491525	-0.044699807	0
0.178575838	Fisher's Exact	0.078497796	1.327875701	0.436271186	-1.196702901	0.748177304
0.073468616	Fisher's Exact	0.529525498	104.4460261	7.271186441	2.862190788	1.133898141
0.113050529	Fisher's Exact	0.40584986	44.21602786	4.847457627	2.277228287	0.946727401
0.250347817	Fisher's Exact	0.238148624	14.22557971	2.423728814	1.277228287	0.601456191
0.250347817	Fisher's Exact	0.238148624	14.22557971	2.423728814	1.277228287	0.601456191
0.681544359	Fisher's Exact	0.129301413	5.690739066	1.211864407	0.277228287	0.166505873
1	Fisher's Exact	0.111900537	4.713586394	1.03874092	0.054835866	0
1	Fisher's Exact	0.083416313	3.277142489	0.765388046	-0.385736725	0
1	Fisher's Exact	0.083416313	3.277142489	0.765388046	-0.385736725	0
0.759313008	Fisher's Exact	0.06349159	2.380166125	0.581694915	-0.781665402	0.11957916
0.045821452	Fisher's Exact	0.029276123	1.016181367	0.279661017	-1.83824893	1.338931157
3.96052E-05	Fisher's Exact	0.012312836	0.412586604	0.135910027	-2.879276198	4.402247967
0.120901639	Fisher's Exact	0.186440643	#NUM!	100000	4*	0.91756781
0.120901639	Fisher's Exact	0.186440643	#NUM!	100000	4*	0.91756781
0.120901639	Fisher's Exact	0.186440643	#NUM!	100000	4*	0.91756781
0.227404315	Fisher's Exact	0.092425553	577.6168657	7.271186441	2.862190788	0.643201298
0.227404315	Fisher's Exact	0.092425553	577.6168657	7.271186441	2.862190788	0.643201298
0.321196796	Fisher's Exact	0.061345772	71.59151912	3.63559322	1.862190788	0.493228796
0.321196796	Fisher's Exact	0.061345772	71.59151912	3.63559322	1.862190788	0.493228796
0.321196796	Fisher's Exact	0.061345772	71.59151912	3.63559322	1.862190788	0.493228796
0.403772856	Fisher's Exact	0.045854053	30.97403774	2.423728814	1.277228287	0.393862881
0.403772856	Fisher's Exact	0.045854053	30.97403774	2.423728814	1.277228287	0.393862881
0.403772856	Fisher's Exact	0.045854053	30.97403774	2.423728814	1.277228287	0.393862881
0.476453437	Fisher's Exact	0.036574395	18.90104189	1.81779661	0.862190788	0.321979536
0.476453437	Fisher's Exact	0.036574395	18.90104189	1.81779661	0.862190788	0.321979536
0.476453437	Fisher's Exact	0.036574395	18.90104189	1.81779661	0.862190788	0.321979536
0.476453437	Fisher's Exact	0.036574395	18.90104189	1.81779661	0.862190788	0.321979536
0.54040633	Fisher's Exact	0.030394352	13.38628719	1.454237288	0.540262693	0.267279573
0.54040633	Fisher's Exact	0.030394352	13.38628719	1.454237288	0.540262693	0.267279573
0.54040633	Fisher's Exact	0.030394352	13.38628719	1.454237288	0.540262693	0.267279573
0.54040633	Fisher's Exact	0.030394352	13.38628719	1.454237288	0.540262693	0.267279573
0.596663646	Fisher's Exact	0.025983164	10.29149716	1.211864407	0.277228287	0.224270422
1	Fisher's Exact	0.018049589	5.995920376	0.807909605	-0.307734213	0
1	Fisher's Exact	0.016367791	5.249689432	0.727118644	-0.459737307	0
1	Fisher's Exact	0.016367791	5.249689432	0.727118644	-0.459737307	0
1	Fisher's Exact	0.016367791	5.249689432	0.727118644	-0.459737307	0
1	Fisher's Exact	0.014969859	4.665117061	0.661016949	-0.59724083	0
1	Fisher's Exact	0.013784432	4.194543092	0.605932203	-0.722771713	0
0.706245858	Fisher's Exact	0.011117937	3.210715593	0.484745763	-1.044699807	0.151044086
0.706245858	Fisher's Exact	0.011117937	3.210715593	0.484745763	-1.044699807	0.151044086
0.710545893	Fisher's Exact	0.009834429	2.770904504	0.427716849	-1.225272053	0.148407866
0.498832371	Fisher's Exact	0.007967934	2.167594018	0.346246973	-1.530126635	0.302045371
0.347178034	Fisher's Exact	0.006676049	1.773258203	0.290847458	-1.781665402	0.45944776
0.156618559	Fisher's Exact	0.005728829	1.495686731	0.250730567	-1.995790207	0.805156777
0.106103457	Fisher's Exact	0.004702834	1.205318261	0.207748184	-2.267092229	0.974270468
0.069157809	Fisher's Exact	0.004432863	1.130649392	0.196518552	-2.347262577	1.160158777
0.030647069	Fisher's Exact	0.003502416	0.878664054	0.15806927	-2.661371168	1.513611051

Supplementary Table 7

Gene	sum_1	length_1	freq1	sum_2	length_2	freq2	Ratio clival/SU2C	statistic/estimate
TP53	4	19	21.05263158	138	424	32.54716981	0.646834477	0.553317519
BRCA2	4	19	21.05263158	23	424	5.424528302	3.881006865	1.553317519
CTNNB1	3	19	15.78947368	22	424	5.188679245	3.043062201	2.553317519
TMPRSS2-ETS	2	19	10.52631579	128	424	30.18867925	0.348684211	3.553317519
PTEN	2	19	10.52631579	103	424	24.29245283	0.4333163	4.553317519
ATM	2	19	10.52631579	18	424	4.245283019	2.479532164	5.553317519
BRAF	2	19	10.52631579	15	424	3.537735849	2.975438596	6.553317519
FAT1	1	19	5.263157895	6	424	1.41509434	3.719298246	7.553317519
BAP1	1	19	5.263157895	1	424	0.235849057	22.31578947	8.553317519
APC	1	19	5.263157895	43	424	10.14150943	0.518971848	9.553317519
CHEK2	1	19	5.263157895	3	424	0.70754717	7.438596491	10.55331752
RB1	1	19	5.263157895	20	424	4.716981132	1.115789474	11.55331752
KDM6A	1	19	5.263157895	6	424	1.41509434	3.719298246	12.55331752
MYC	1	19	5.263157895	30	424	7.075471698	0.743859649	13.55331752
PIK3CA	1	19	5.263157895	18	424	4.245283019	1.239766082	14.55331752
PALB2	1	19	5.263157895	0	424	0	#DIV/0!	15.55331752
BRCA1	1	19	5.263157895	1	424	0.235849057	22.31578947	16.55331752
ERCC2	1	19	5.263157895	1	424	0.235849057	22.31578947	17.55331752
SMARCA4	1	19	5.263157895	1	424	0.235849057	22.31578947	18.55331752
MLH1	1	19	5.263157895	1	424	0.235849057	22.31578947	19.55331752
FGFR2	1	19	5.263157895	0	424	0.235849057	22.31578947	20.55331752
AR	0	19	0	23	424	5.424528302	0	0

*adjusted Log2FC

Supplementary Table 7

p.value	method	conf.low	conf.high	FC	Log2_FC	Log10_p
0.451013589	Fisher's Exact Tes	0.131200239	1.780310048	0.646834477	-0.628531516	0.345810372
0.022613221	Fisher's Exact Tes	1.033860836	16.15239481	3.881006865	1.956430985	1.645637582
0.084403417	Fisher's Exact Tes	0.593310144	13.2776725	3.043062201	1.605523823	1.07363997
0.073928199	Fisher's Exact Tes	0.030125094	1.175598146	0.348684211	-1.520007059	1.131189873
0.267987474	Fisher's Exact Tes	0.040496037	1.589109729	0.4333163	-1.206507586	0.571885505
0.209228133	Fisher's Exact Tes	0.2759823	12.61640089	2.479532164	1.31006794	0.679379921
0.161539302	Fisher's Exact Tes	0.328979146	15.61480857	2.975438596	1.573102346	0.791721799
0.265819177	Fisher's Exact Tes	0.07976173	34.43363338	3.719298246	1.89503044	0.575413692
0.084032154	Fisher's Exact Tes	0.283716475	1816.17458	22.31578947	4.479992941	1.075554503
0.70846219	Fisher's Exact Tes	0.011552756	3.279429499	0.518971848	-0.946271814	0.149683323
0.161344674	Fisher's Exact Tes	0.140765217	101.3017976	7.438596491	2.89503044	0.792245365
0.610347403	Fisher's Exact Tes	0.025667499	7.865212473	1.115789474	0.158064846	0.214422899
0.265819177	Fisher's Exact Tes	0.07976173	34.43363338	3.719298246	1.89503044	0.575413692
1	Fisher's Exact Tes	0.01695414	4.9531859	0.743859649	-0.426897654	0
0.572882456	Fisher's Exact Tes	0.028511495	8.877808489	1.239766082	0.31006794	0.241934477
0.042889391	Fisher's Exact Tes	0.572194733	#NUM!	1000000	5*	1.367650125
0.084032154	Fisher's Exact Tes	0.283716475	1816.17458	22.31578947	4.479992941	1.075554503
0.084032154	Fisher's Exact Tes	0.283716475	1816.17458	22.31578947	4.479992941	1.075554503
0.084032154	Fisher's Exact Tes	0.283716475	1816.17458	22.31578947	4.479992941	1.075554503
0.084032154	Fisher's Exact Tes	0.283716475	1816.17458	22.31578947	4.479992941	1.075554503
0.042889391	Fisher's Exact Tes	0.572194733	#NUM!	22.31578947	4.479992941	1.367650125
0.613749094	Fisher's Exact Tes	0	4.033742573	0	-5*	0.212009136

Supplementary Table 8

	Clival metastasis			SU2C					
**Overall**	**altered**	**total**	**altered**	**no**	**total**	**freq**	**Ratio clival/SU**	**statistic**	**p.value**
Homologous Recombinant Repair	26	59	44.06779661	110	429	25.64102564	1.718644068	7.868165476	0.005031267
AR pathways	26	59	44.06779661	305	429	71.0955711	0.619838844	16.14640494	5.86297E-05
p53 Pathway	23	59	38.98305085	229	429	53.37995338	0.730293835	3.747622248	0.052882689
UV Response Dn	10	59	16.94915254	34	429	7.925407925	2.138584247	4.107099331	0.042703544
Wnt-beta Catenin Signaling	10	59	16.94915254	58	429	13.51981352	1.253652835	0.262858179	0.608163312
PI3K/AKT/mTOR Signaling	10	59	16.94915254	179	429	41.72494172	0.406211533	12.3930987	0.000430924
G2-M Checkpoint	10	59	16.94915254	199	429	46.38694639	0.365386253	17.17390566	3.4109E-05
RAF kinases	6	59	10.16949153	12	429	2.797202797	3.63559322	5.995727065	0.01434057
Mismatch Repair Pathway	3	59	5.084745763	12	429	2.797202797	1.81779661	1.858600452	0.408294446
**CRPC clival mets**	**altered**	**total**	**altered**	**no**	**total**	**freq**	**Ratio clival/SU**	**statistic**	**p.value**
Homologous Recombinant Repair	17	40	42.5	110	429	25.64102564	1.6575	4.447326983	0.034955921
AR pathways	19	40	47.5	305	429	71.0955711	0.668114754	8.464821125	0.003620809
p53 Pathway	19	40	47.5	229	429	53.37995338	0.889847162	0.299126197	0.584430736
UV Response Dn	6	40	15.0	34	429	7.925407925	1.892647059	1.528083746	0.216400268
Wnt-beta Catenin Signaling	6	40	15.0	58	429	13.51981352	1.109482759	0.00040095	0.984024446
PI3K/AKT/mTOR Signaling	6	40	15.0	179	429	41.72494172	0.359497207	9.850164117	0.001698166
G2-M Checkpoint	6	40	15.0	199	429	46.38694639	0.323366834	13.40186317	0.000251374
RAF kinases	4	40	10.0	12	429	2.797202797	3.575	3.842467214	0.039002797
Mismatch Repair Pathway	2	40	5.0	12	429	2.797202797	1.7875	1.825927391	0.338819027

Supplementary Table 8

method
Pearson's Chi-squared test with Yates' continuity correction
Pearson's Chi-squared test with Yates' continuity correction
Pearson's Chi-squared test with Yates' continuity correction
Pearson's Chi-squared test with Yates' continuity correction
Pearson's Chi-squared test with Yates' continuity correction
Pearson's Chi-squared test with Yates' continuity correction
Pearson's Chi-squared test with Yates' continuity correction
Pearson's Chi-squared test with Yates' continuity correction
Fisher's Exact Test for Count Data
**method**
Pearson's Chi-squared test with Yates' continuity correction
Pearson's Chi-squared test with Yates' continuity correction
Pearson's Chi-squared test with Yates' continuity correction
Pearson's Chi-squared test with Yates' continuity correction
Pearson's Chi-squared test with Yates' continuity correction
Pearson's Chi-squared test with Yates' continuity correction
Pearson's Chi-squared test with Yates' continuity correction
Fisher's Exact Test for Count Data
Fisher's Exact Test for Count Data
