# Supplementary material for: Molecular landscape of prostate cancers with clival metastases
Source: Oncologist. 2026 Mar 4;31(4):oyag074. doi: 10.1093/oncolo/oyag074 (PMC12995431; doi:10.1093/oncolo/oyag074)

Supplementary Figures

Supplementary Figure 1:

OncoPrint figure summarizing genomic alterations among all patients with mCRPC clival metastases (n = 40). The top and right panels display bar plots indicating the number and types of alterations per patient and per gene, respectively. The left panel shows the percentage of samples harboring alterations in each gene. The bottom panel depicts the number of samples contributed by each patient. Tissue types are color-coded: tissues (purple), blood (pink), and both (blue). * Denotes neuroendocrine carcinoma; the rest were adenocarcinoma.

Supplementary Figure 2

A. Volcano plot comparing alteration frequencies between the clival metastasis cohort (n=59) and the SU2C mCRPC cohort (n=429). The x-axis represents the $\log_2$ fold change in alteration frequency for each gene, and the y-axis shows the $-\log_{10}$ p-value. Colors denote pathway classifications. The dashed horizontal line represents a p-value of 0.05.

B. Bar graphs depicting the proportion of gene alterations in clival metastases (n=59, blue) compared with the SU2C mCRPC cohort (n=429, gold)

C. Bar graphs depicting the proportion of patients in the clival metastasis cohort and the SU2C mCRPC cohort with alterations in different pathways. Pathways were defined as in Figure 3.

Supplementary Figure 3

A. OncoPrint figure summarizing genomic alterations among all patients with HSPC clival metastases (n = 19). The top and right panels display bar plots indicating the number and types of alterations per patient and per gene, respectively. The left panel shows the percentage of samples harboring alterations in each gene. The bottom panel depicts the number of samples contributed by each patient. Tissue types are color-coded: tissues (purple) and blood (pink). * Denotes samples with NGS data at or after the time of clival metastases.

B. Volcano plot comparing alteration frequencies between patients with HSPC clival metastases cohort and the mHSPC cohort (CCR 2020). The x-axis represents the $\log_2$ fold change in alteration frequency for each gene, and the y-axis shows the $-\log_{10}$ p-value. Colors denote pathway classifications. The dashed horizontal line represents a p-value of 0.05.

Supplementary Figure 4

A. OncoPrint Figure summarizing genomic alterations in patients with clival metastases who had sequencing at the time of clival metastases (n = 33). The top and right panels display bar plots indicating the number and types of alterations per patient and per gene, respectively. The left panel shows the percentage of samples harboring alterations in each gene. The bottom panel depicts the number of samples contributed by each patient. Tissue types are color-coded: tissues (purple), blood (pink), and both (blue). * Denotes neuroendocrine carcinoma; the rest were adenocarcinoma.

B. Volcano plot comparing alteration frequencies between patients with clival metastases who had sequencing at the time of clival metastases and the SU2C cohort. The x-axis represents the $\log_2$ fold change in alteration frequency for each gene, and the y-axis shows the $-\log_{10}$ p-value. Colors denote pathway classifications. The dashed horizontal line represents a p-value of 0.05.

Supplementary Figure 1.

A

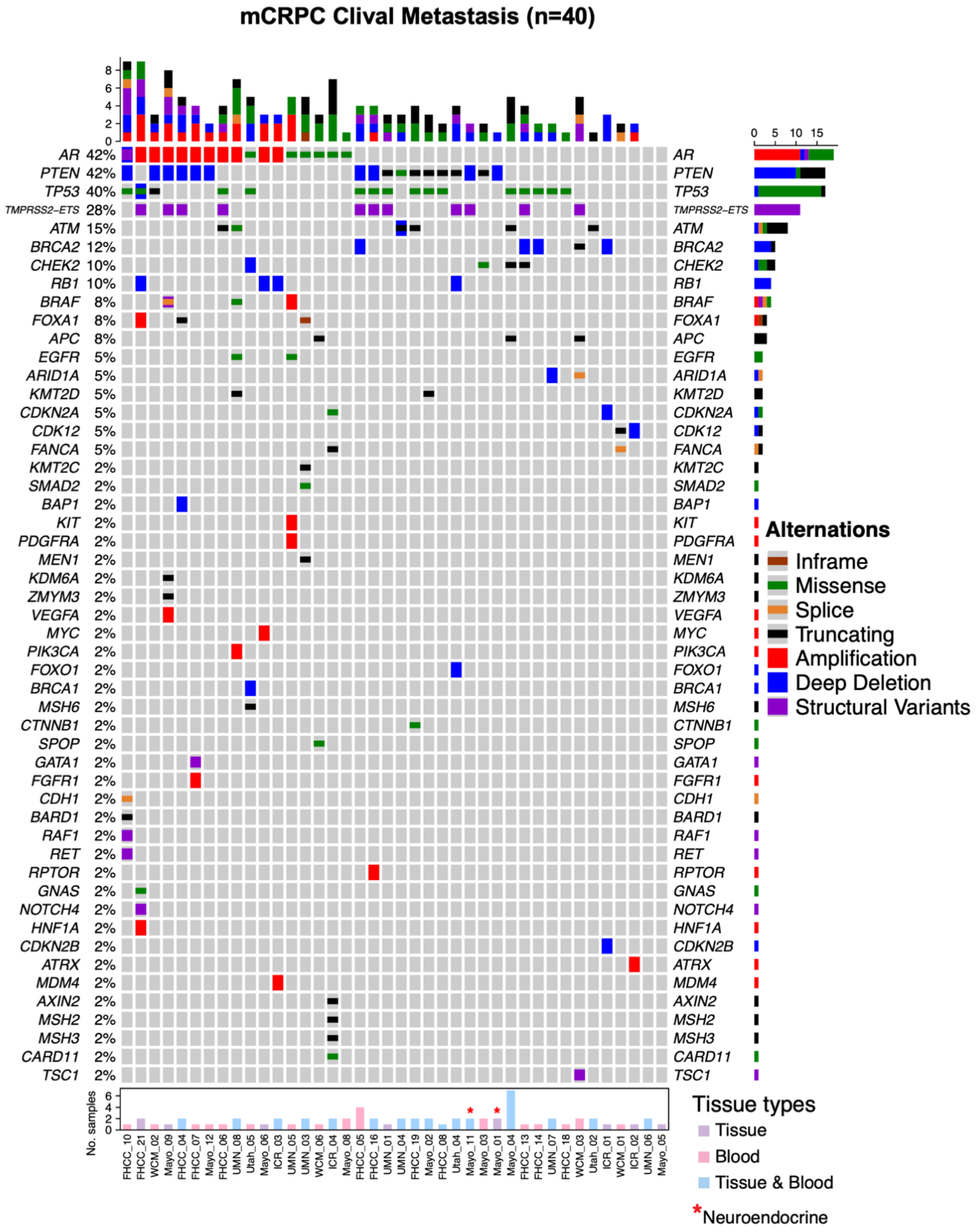

Supplementary Figure 2.

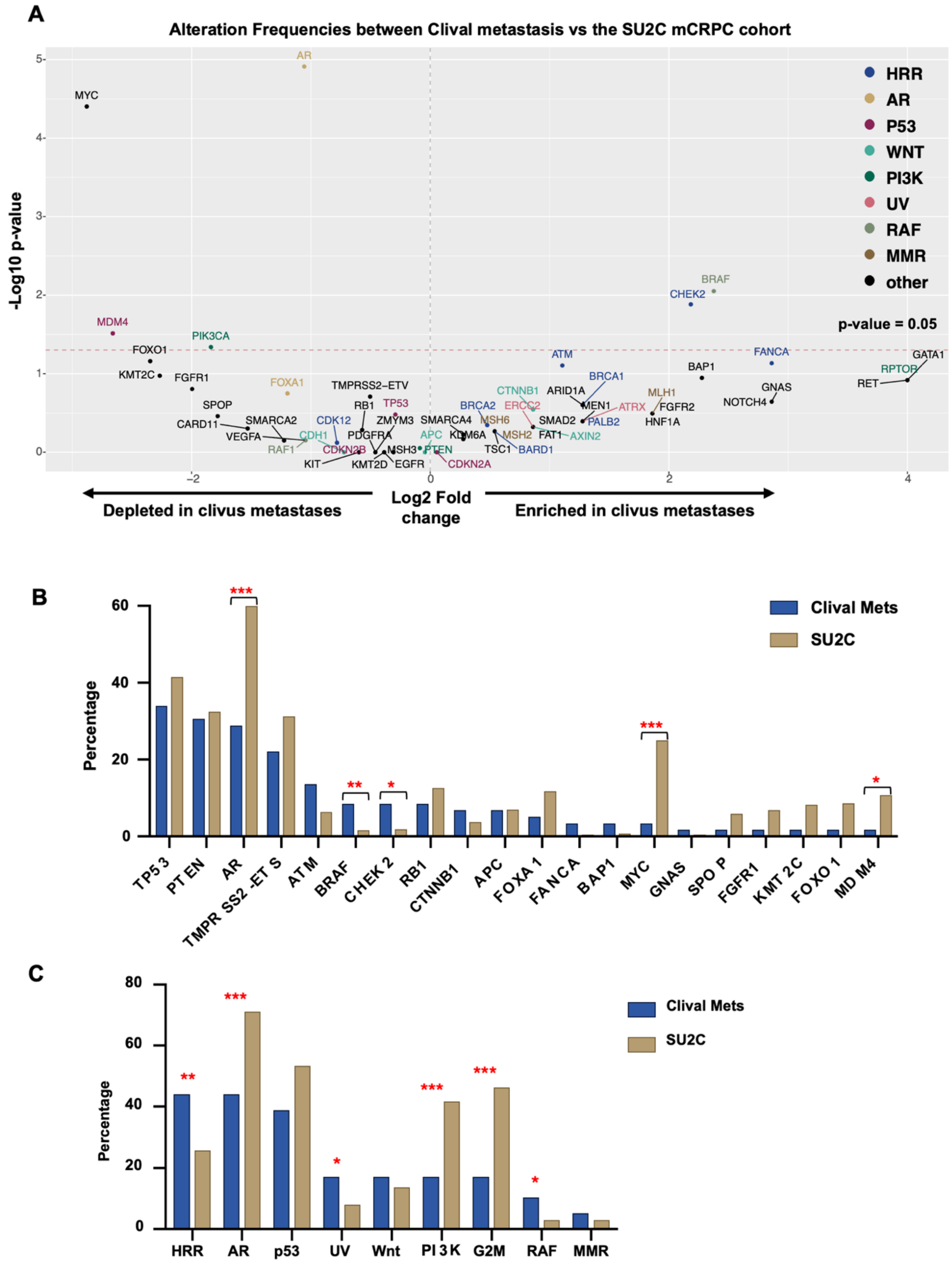

Supplementary Figure 3.

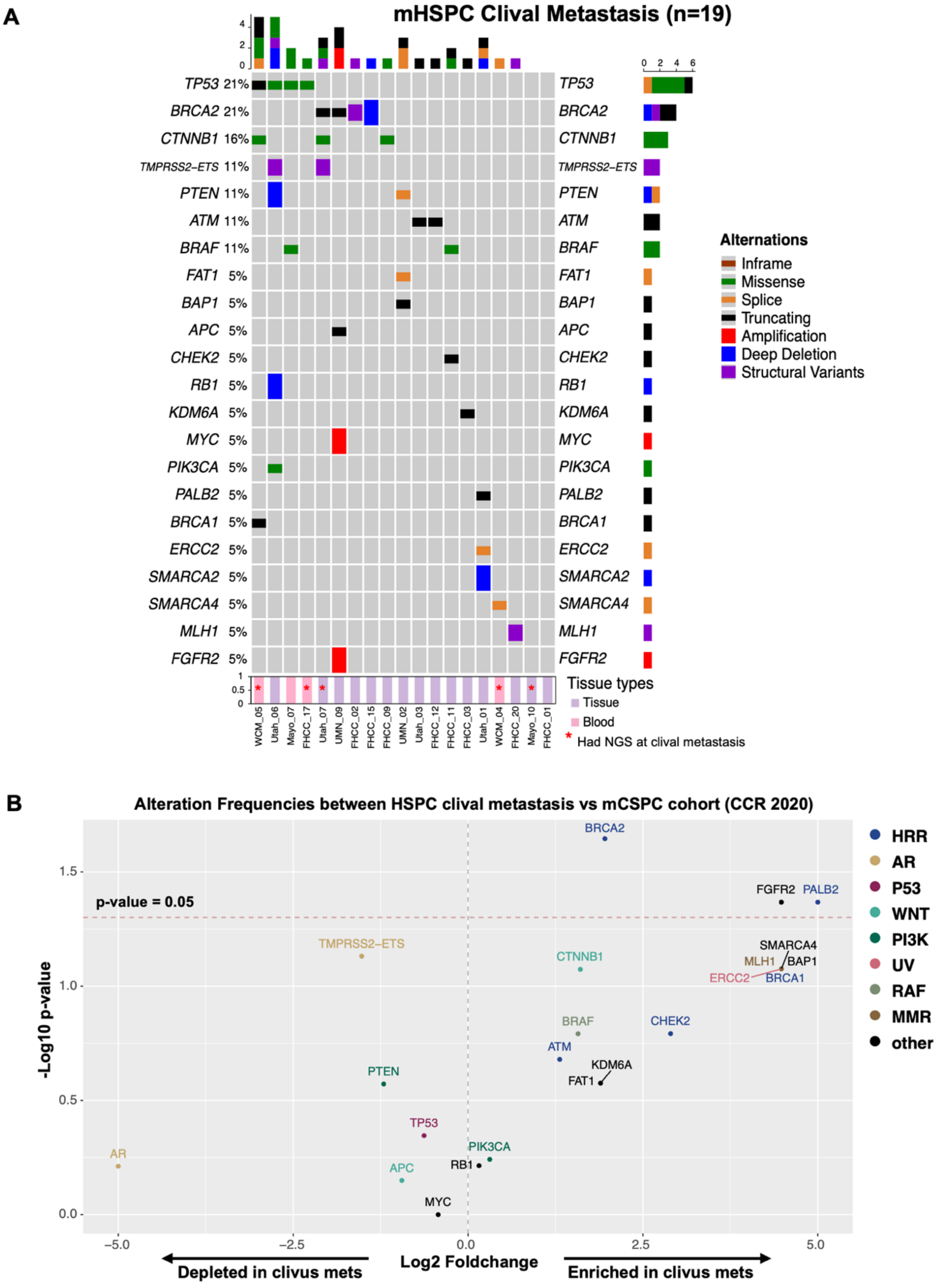

Supplementary Figure 4.

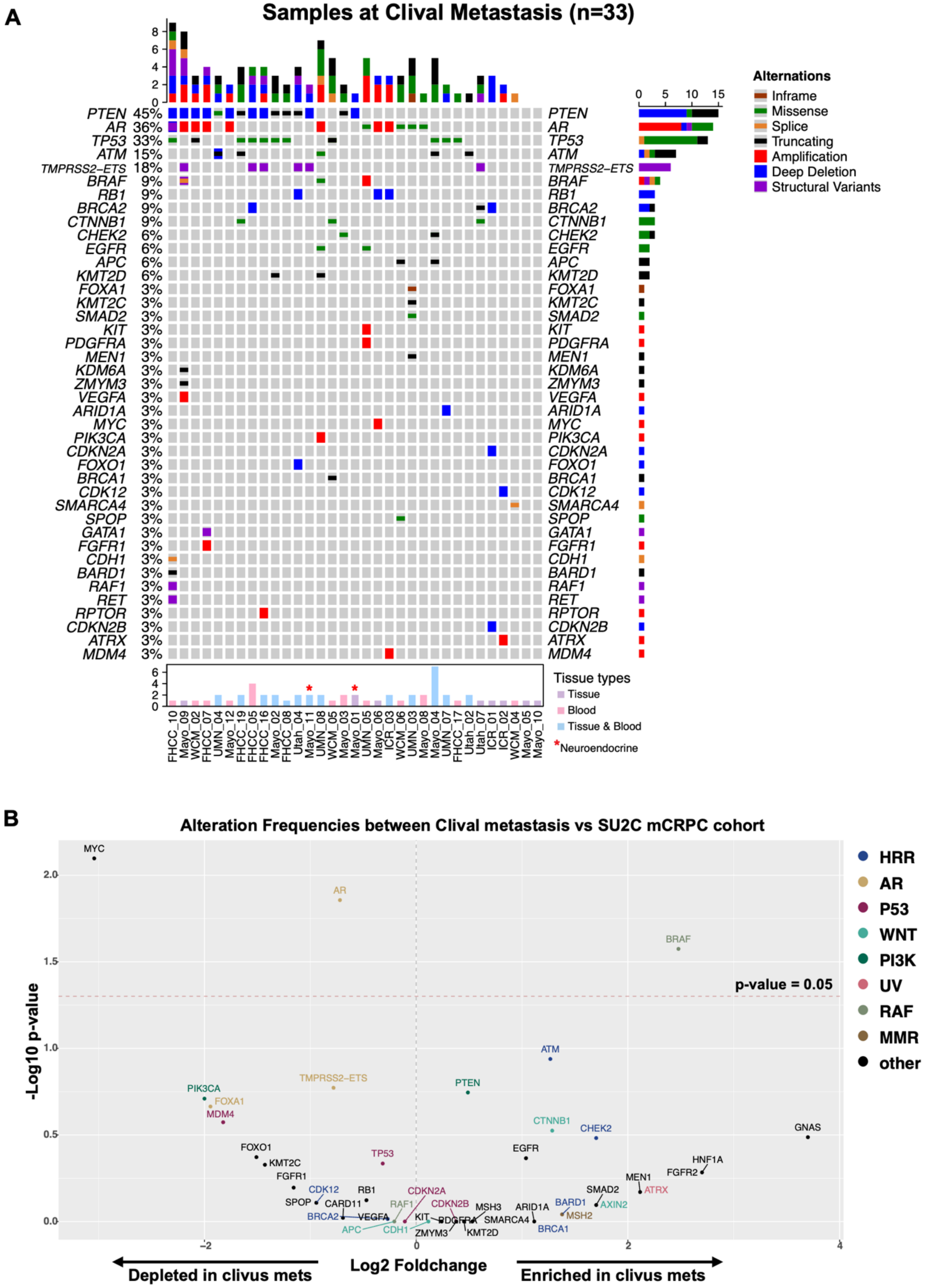

Supplement: oyag074_Supplementary_Data [file oyag074_supplementary_data.zip › SupplementaryFigures-20260210.pdf]
